# Printed Stretchable Graphene Conductors for Wearable Technology

**DOI:** 10.1021/acs.chemmater.2c02007

**Published:** 2022-08-29

**Authors:** Laura S. van Hazendonk, Artur M. Pinto, Kirill Arapov, Nikhil Pillai, Michiel R. C. Beurskens, Jean-Pierre Teunissen, Asko Sneck, Maria Smolander, Corne H. A. Rentrop, Piet C. P. Bouten, Heiner Friedrich

**Affiliations:** † Laboratory of Physical Chemistry and Center for Multiscale Electron Microscopy, Department of Chemical Engineering and Chemistry, Eindhoven University of Technology, P.O. Box 513, 5600MB Eindhoven, The Netherlands; ‡ LEPABE, Faculdade de Engenharia, Universidade do Porto, 4200-180 Porto, Portugal; § Pulseforge, 400 Parker Drive, Suite 1110, Austin, Texas 78728, United States; ∥ Holst Centre - TNO, High Tech Campus 31, 5656AE Eindhoven, The Netherlands; ⊥ VTT Technical Research Centre of Finland Ltd., P.O. Box 1000, FI-02044 Espoo, Finland; # Institute for Complex Molecular Systems, Department of Chemical Engineering and Chemistry, Eindhoven University of Technology, P.O. Box 513, 5600MB Eindhoven, The Netherlands

## Abstract

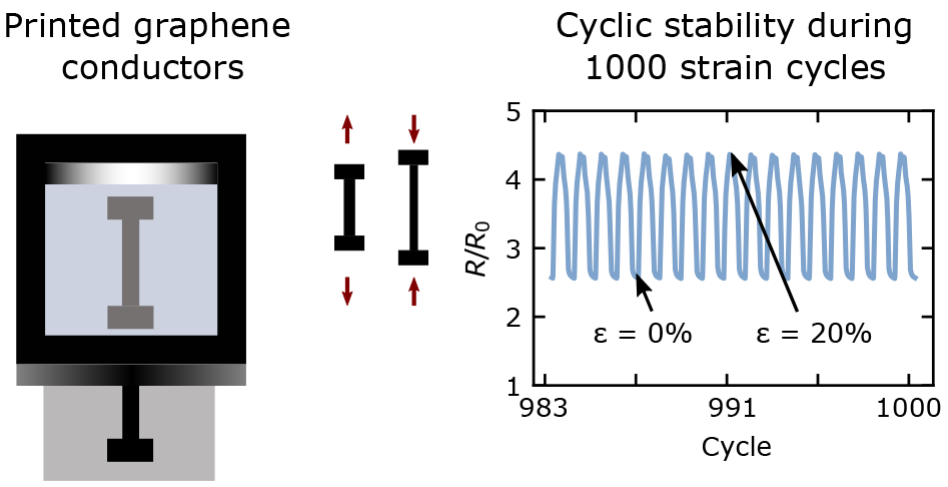

Skin-compatible printed stretchable conductors that combine a low gauge factor with a high durability over many strain cycles are still a great challenge. Here, a graphene nanoplatelet-based colloidal ink utilizing a skin-compatible thermoplastic polyurethane (TPU) binder with adjustable rheology is developed. Stretchable conductors that remain conductive even under 100% strain and demonstrate high fatigue resistance to cyclic strains of 20–50% are realized via printing on TPU. The sheet resistances of these conductors after drying at 120 °C are as low as 34 Ω □^–1^ mil^–1^. Furthermore, photonic annealing at several energy levels is used to decrease the sheet resistance to <10 Ω □^–1^ mil^–1^, with stretchability and fatigue resistance being preserved and tunable. The high conductivity, stretchability, and cyclic stability of printed tracks having excellent feature definition in combination with scalable ink production and adjustable rheology bring the high-volume manufacturing of stretchable wearables into scope.

## Introduction

Printing of conductors has emerged as a more sustainable, flexible, and cost-effective alternative to traditional manufacturing techniques, as material is deposited only where needed, thus minimizing waste.^1^ Printing also enables the scalable production of flexible electronics that are tolerant to mechanical bending and/or stretching.^2−4^ This facilitates the manufacturing of wearable electronics, which show great potential for medical monitoring applications and for the sports industry.^5^ Wearable power sources,^6^ supercapacitors,^7,8^ (biomedical) sensors,^9−11^ and e-textiles^12^ have already emerged in the scientific realm. At present, the conductive components of printed electronics are often composed of metals. Metals are, however, prone to electromigration and scarce, and while silver and gold are highly expensive, copper is toxic and sensitive to oxidation.^2,13,14^ Alternatives to metals that would be ideal for integration into wearable conductors are flexible conductive polymers, but they suffer from stability issues.^15^ An even better solution would be the carbon allotrope graphene, which is environmentally inert, mechanically strong, abundant, and highly conductive.^2,15−17^ Importantly for wearable applications, it has been classified as a low irritant on skin.^18^ Because of developments in liquid-phase exfoliation (LPE), graphene nanoplatelets (GNPs) can be produced relatively cheaply and in large volumes, after which ink production is a natural next step.^19,20^ This makes inks based on GNPs an attractive complement to their metal-based counterparts for wearable technology.

A variety of industrial printing approaches exist, each demanding inks with different properties such as rheology, surface tension, and drying time.^21^ Inkjet printing is ideal for high-resolution deposition but requires inks of low viscosity, and hence low concentration, limiting the conductivity of printed tracks. In contrast, flexographic and screen printing technologies offer a simple, flexible, fast, and industrially scalable method for producing wearable electronics.^15,21,22^ In particular, screen printing is highly compatible with a wide range of inks and substrates and prints thick layers, enabling relatively low resistances.

GNP-based inks suitable for screen printing of flexible conductors with applications in sensors, photovoltaics, and wireless communications have already been demonstrated.^22−33^ Beyond flexibility, many applications require stretchability.^3,4,15,34^ The main difference between flexible and stretchable printed electronics is the strain level reached in each case, which is orders of magnitude higher in stretchable electronics. This is essential for applications such as athletic garments,^35^ on-body sensors,^33,35^ sensory artificial skin,^36^ wearable energy storage devices,^8^ stretchable light-emitting diodes (LEDs),^36^ soft robotics,^37,38^ strain sensors,^39−42^ and cardiac implants.^37^ Furthermore, stretchability is expected to generally improve the lifetime of flexible electronic devices by reducing fatigue^43^ and enables conformal printing to nonflat, flexible substrates.^44^

Lately, graphene-based strain sensors have been manufactured through screen printing.^45−47^ In addition, stretchable supercapacitors were printed from inks composed of a conductive polymeric binder (PEDOT:PSS) to which some graphene was added to enhance the performance.^8^ Recently, printed stretchable sweat sensors were realized from an ink containing GNP and a thermoplastic polyurethane (TPU) binder in *N*-methyl-2-pyrrolidone (NMP),^33^ and strain sensors were produced by decorating cotton fabrics with a GNP-based ink followed by a polyurethane layer.^42^ Although these inks offer significant progress, additional strategies are needed to realize graphene-based inks with adjustable rheology, to increase the conductivity of printed tracks, and to preserve stretchability over many cycles.

Ideally, conductive tracks in wearable electronics have a high conductivity even after a single print pass, high stretchability, and a low gauge factor,^2,3,48^ which is defined as the relative increase in resistance in response to strain.^49^ Such conductors would then be exposed to repetitive strains of 20–50%, corresponding to the stretchability of the skin in different regions of the body.^1,15,33−35,50−52^ To offer a realistic chance of adoption into wearables, these stretchable conductors must be printable from a GNP-based ink formulated with environmentally friendly and skin-compatible chemicals. The ink should have a rheology that can be adjusted to the additive manufacturing process of choice, e.g., screen printing, and offer reasonably high print definition on stretchable substrates. Furthermore, the ink production process should be scalable. To the best of our knowledge, an ink that fulfills all of these requirements does not yet exist.

In this study, we present a GNP-based ink that meets all of the requirements mentioned above for screen printing of skin-compatible, stretchable, and durable conductors with low sheet resistances of 34 Ω □^–1^ mil^–1^ on thermoplastic polyurethane (TPU) substrates after drying at 120 °C, which can be further improved to <10 Ω □^–1^ mil^–1^ while preserving the stretchability by means of post-treatment with photonic annealing. This approach extends our scalable production method for GNPs^20^ on flexible substrates,^23^ now using a stretchable and skin-compatible TPU binder system that facilitates an adjustable rheology. This GNP ink yields straight conductors that remain conductive even at 100% strain. Cyclic straining for 1000 cycles at 20–50% strain demonstrates that the conductors combine a low gauge factor with minimal drift (fatigue) over time. Furthermore, via postprocessing by photonic annealing, the resistance, gauge factor and drift can be tuned without compromising the stretchability of the flexible substrates. This work opens a route toward the scalable production of skin-compatible wearables such as motion sensors, heart rate monitors, athletic garments, and artificial skin.

## Results and Discussion

### Ink Formulation, Rheology, and Printing

**Figure 1:**
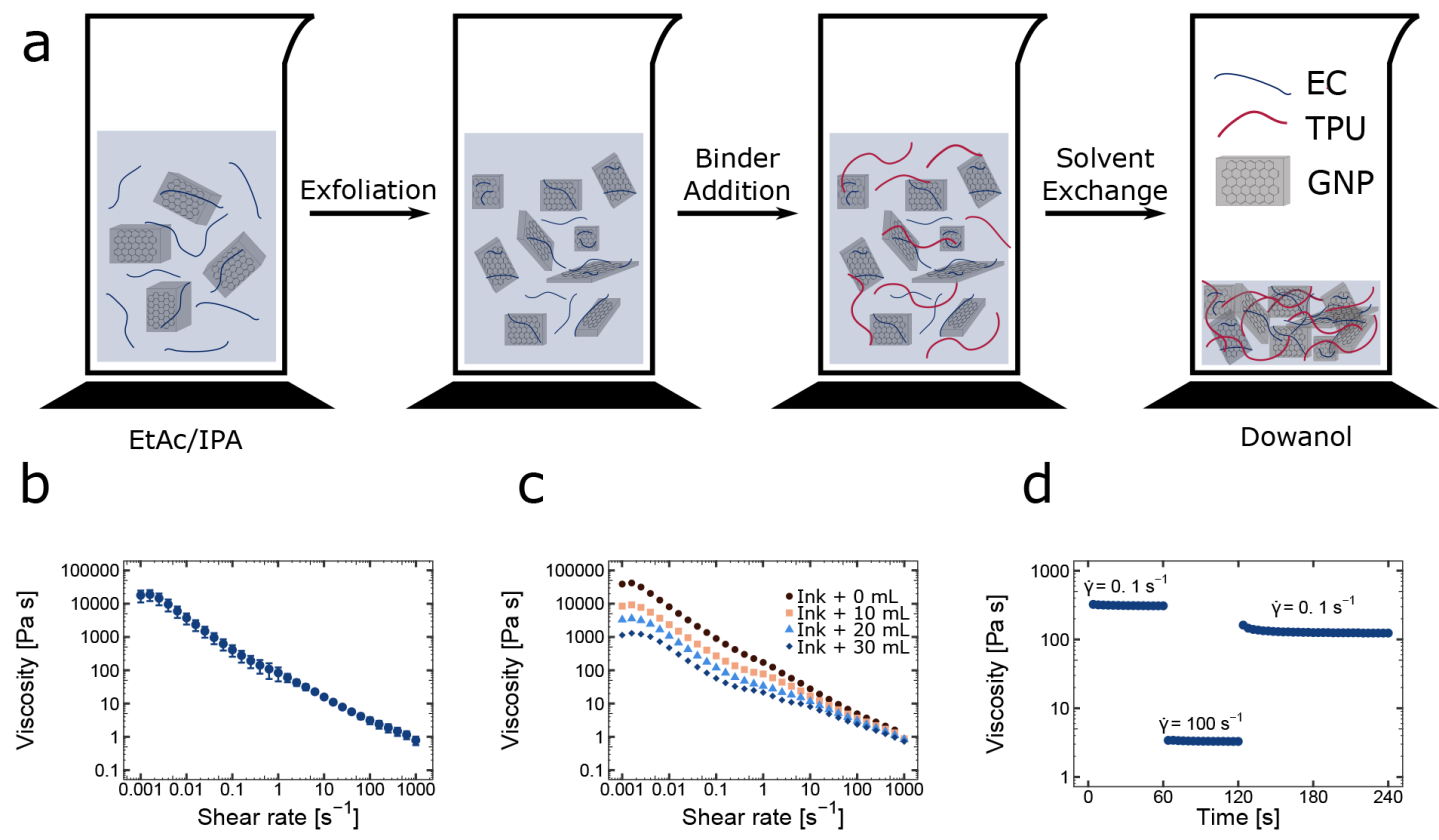
Ink preparation and rheology. (a) Scheme of exfoliation of expanded graphite in ethyl acetate (EtAc) and isopropyl alcohol (IPA) in the presence of ethyl cellulose (EC) to produce graphene nanoplatelets (GNPs), followed by addition of a TPU binder and solvent exchange for propylene glycol *n*-butyl ether (Dowanol). Viscosity flow ramps of (b) five replicate GNP inks (average ± standard deviation multiplied by a factor 2 for visibility) and (c) a serial dilution of one ink with 10 mL steps. (d) Peak-hold test on a typical ink from panel b where shear rate γ̇ is varied from 0.1 to 100 s^–1^ and back to emulate the screen printing process.

To meet the goal of screen printable stretchable conductors for application in wearable electronics, we set three requirements for the ink formulation. First, the GNP concentration must be sufficiently high and the platelet size large enough to ensure a conductivity adequate for the target application. Second, safe solvents must be used to avoid accidental exposure of residual toxins or irritants to the human skin. Third, the ink’s polymeric binders must support stretchability and substrate adhesion to, e.g., TPU. Figure 1a summarizes the ink production process. We employed a scalable production method with low-toxicity compounds with a maximum Chemwatch toxicity rating of 1 on a scale of 1–4. High-shear mixing was selected for LPE of graphite as it constitutes a scalable exfoliation method.^53^ However, the concentrations obtained with high-shear exfoliation are generally rather low, in particular in nontoxic solvents. To achieve a suitable GNP loading and relatively large flake sizes, exfoliation was preceded by graphite pretreatment with sulfuric acid intercalation, washing, and thermal expansion as previously described and detailed in section 1 of the Supporting Information (Figures S1–S3).^20,23^ Exfoliation took place in a mild solvent blend of ethyl acetate (EtAc) and isopropyl alcohol (IPA) (4:1). Ethyl cellulose (EC) was added to improve the exfoliation efficiency.^54,55^ Next, the skin-compatible TPU binder was incorporated and the exfoliation solvent was exchanged^23^ for propylene glycol ethers with a higher boiling point suitable for screen and flexographic printing. The exfoliation solvent (EtAc/IPA) may be subsequently recycled for the next batch of ink. For both screen printing and blade coating applications, the ink was composed of 4.3 wt % GNP, 12.8 wt % TPU, 0.4 wt % EC, and 82.5 wt % solvent; further details are provided in the Experimental Section. The resulting inks are generally stable for ≤1 month, after which phase separation between the GNP-TPU network in one phase and the solvent in another gradually occurs. Homogenization is therefore recommended before printing.

The inks prepared in the manner described above display shear-thinning behavior (Figure 1b). This is essential during screen printing, as the ink must flow easily through the mesh, after which the initial high viscosity should be recovered to maintain feature definition.^21,56^ This shear-thinning behavior represents an inherent property of two-dimensional (2D) colloidal systems that align under shear and can thus be extended to other 2D crystal inks.^57,58^ The capability of the ink to recover after shearing was investigated by emulating the screen printing process with a peak-hold or three-interval thixotropy (3ITT) test.^29^ In this experiment, after an initial static interval of 60 s with a constant low shear rate γ̇ of 0.1 s^–1^, the shear rate was increased to 100 s^–1^ for 60 s, mimicking the flow through the screen. Finally, the shear rate was maintained at 0.1 s^–1^ for 120 s. The result depicted in Figure 1d indicates an almost instantaneous recovery of the viscosity after removal of shear, which is ideal for ensuring excellent feature definition during the printing process. We hypothesize this fast recovery might be attributed to the viscoelastic nature of the GNP-based inks. As shown in section 2 of the Supporting Information (Figure S4), the ink behaves like a viscoelastic solid even at high angular frequencies, indicative of the presence of a jammed network of platelets in which energy is stored in the form of interparticle interactions.^56,58^ Note that some ink spill was reported during the high-shear interval, such that the final viscosity is slightly lower than the initial one.^29^ To verify the reproducibility of our ink production protocol, the same ink was prepared five times. The rheology curve in Figure 1b indicates excellent reproducibility with a very low standard deviation across inks. To make this ink suitable for a range of screen printing, blade coating, or flexographic printing setups, the viscosity can be adjusted by stirring in additional solvent as shown in Figure 1c. For ink deposition, we first utilized blade coating, also known as doctor blading, on glass substrates as a quick indicator of spreading behavior, layer uniformity, dry layer thickness, and sheet resistance. Following these experiments, the average sheet resistance was 38 Ω □^–1^ mil^–1^ before any postprocessing except for drying at 100 °C for 1 h (Table S1 in section 3 of the Supporting Information), which may be considered already competitive with other GNP- and binder-based ink formulations containing LPE GNP.^21,33,42,46^ Furthermore, the standard deviation across the five inks was only 6 Ω □^–1^ mil^–1^, corroborating the excellent reproducibility of the conductive properties using the ink preparation and deposition described above.

Screen printing experiments were carried out with a semiautomated screen print setup and a 200 mesh metal screen with a minimum feature size of 200 μm as detailed in the Experimental Section (section 4 of the Supporting Information and Figure S5). After some initial printing trials, 80 mm s^–1^ was selected as an appropriate print speed in combination with a print gap of 1.6 mm based on the judgment of an experienced screen printer operator. Under these conditions, the ink was observed to roll on the screen in front of the TPU squeegee, which is an indication of high ink printability. The ink was printed with a single pass on three types of substrates with a range of surface energies and roughness values to show the generality of the GNP-based ink. The selected substrates were polyethylene terephthalate (PET ST504, DuPont), a standard substrate for printed electronics, and two types of stretchable thermoplastic polyurethane (TPU) substrates, EU94 (DelStar Technologies) and ST604 (BEMIS). Visual inspection of feature definition (Figure S5) and wetting behavior showed excellent results confirming the choice of printing conditions and the generality of the ink.

To analyze the quality of the single-pass printed conductors, the dry layer thickness and sheet resistance of dogbone test structures of 76 mm × 1 mm (Figure S6 in section 4 of the Supporting Information) were characterized (Table 1). As the printed conductors were fairly rough due to a combination of large GNPs with a rough substrate, the baseline thickness was selected as an indicator of the active thickness of the conductive path according to an algorithm detailed in section 5 of the Supporting Information (Figure S7 and Table S2). Notably, the sheet resistances are highly competitive^21,33,42,46^ for printed graphene-based conductors with values of 30, 34, and 62 Ω □^–1^ mil^–1^ for tracks printed on PET, TPU EU94, and TPU ST604, respectively (Table 1). Note that resistance values differed slightly between print geometries, as detailed in Table S2. Interestingly, the layers printed on ST604 were thicker than those printed on EU94 and PET ST504. These observations highlight that results are determined by the interplay among the printing equipment, ink, and substrate. A simple scotch tape test indicated decent abrasion resistance for tracks on all substrates (Figure S8 in section 6 of the Supporting Information). To verify the flexibility of the ink formulation for applications beyond screen printing, we performed flexographic printing tests on paper as detailed in section 7 of the Supporting Information. Continuous lines were successfully printed with <100 μm width (Figure S9), which confirms the ink’s high potential for printing applications beyond screen printing.

**Table 1:** Sheet Resistances Normalized to 25 μm and Baseline Thicknesses of Screen-Printed Conductors on TPU EU94/ST604 and PET ST504 Substrates (theoretical wet layer thickness of 43–55 μm)^a^

substrate	*R*_s_ (Ω □^–1^ mil^–1^)	thickness (μm)
TPU EU94	34 ± 2.4	5.8 ± 1.2
TPU ST604	62 ± 3.7	12 ± 1.0
PET ST504	30 ± 2.6	5.7 ± 0.47

a *N* = 10. Errors represent printing standard deviations within one ink.

### Straining Tests of Conductive Tracks

**Figure 2:**
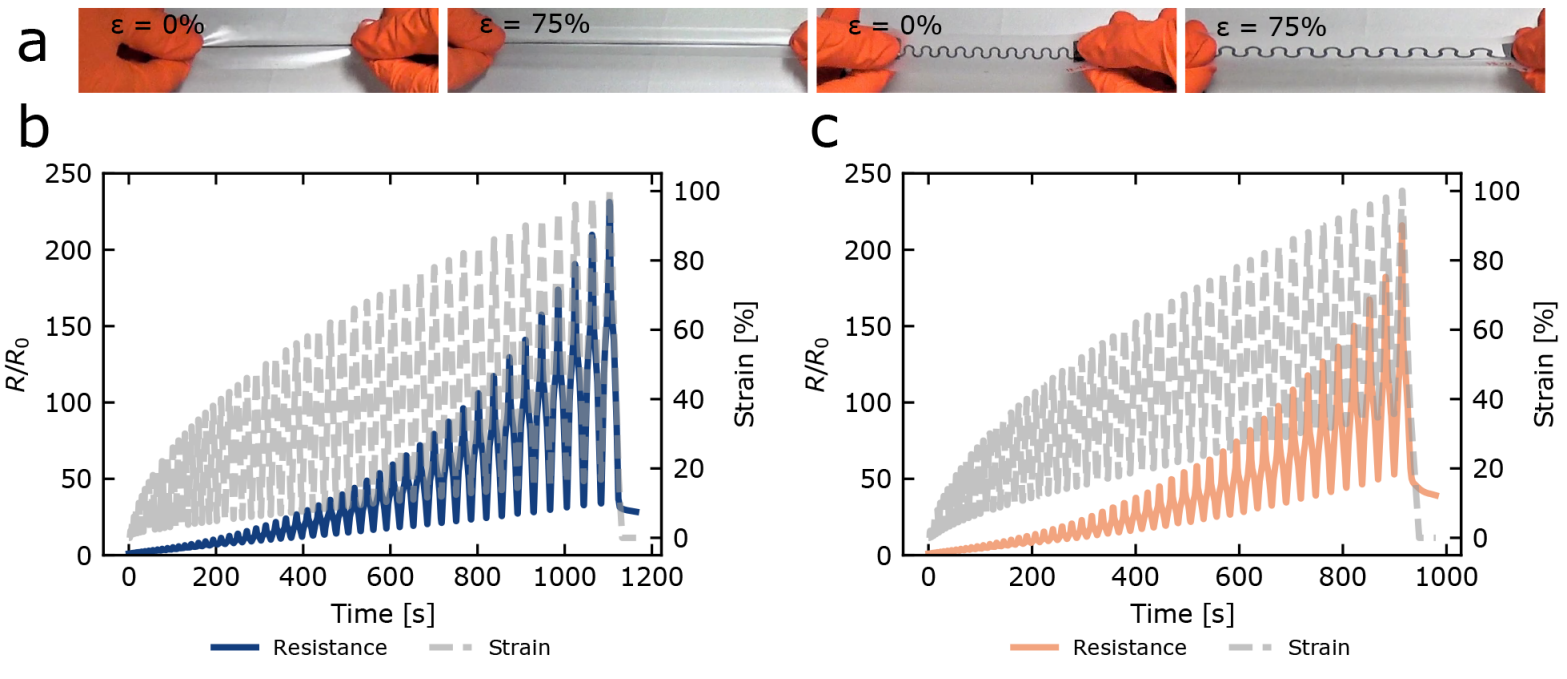
Stretchability of printed conductors. (a) Manual straining of straight and meandering structures (line width of 1 mm) on ST604 up to 75% strain. Electromechanical characterization of straight conductors exposed to strains with amplitudes linearly increasing from 2% to 100% in 50 steps on (b) EU94 and (c) ST604. Corresponding gauge factors are included in Table S3.

Printed conductors suitable for wearable electronics applications on the skin must be able to endure repetitive strains of 20–50%.^15,34,35,50−52^ Manual straining tests shown in Figure 2a looked very promising. To quantify the change in resistance, a Mark-10 straining apparatus equipped with a Keithley System SourceMeter (four-point measurement) and a Leica optical microscope was used (Figure S10 in section 8 of the Supporting Information). First, 1 mm wide conductors (Figure S6) were subjected to a strain test with the strain amplitude increasing from 0% to 100% with increments of 2% strain and a strain rate of 200 mm min^–1^ (Figure 2b,c). While the resistance increased with strain level, the straight tracks remained conductive even at 100% strain. The strain tests with an increasing strain amplitude indicated few macroscopic changes in morphology at strain amplitudes of ≤20%, while the introduction of cracks was initiated only at higher strain levels, which can be distinguished with an optical microscope during straining (Figure S11 and section 9 of the Supporting Information). Second, because cyclic durability is a key requirement for wearables including stretchable conductors, the fatigue behavior of the printed conductors was investigated in detail. As the skin’s stretchability is generally 20%,^35,50,51^ this strain level was selected for cyclic tests.

**Figure 3:**
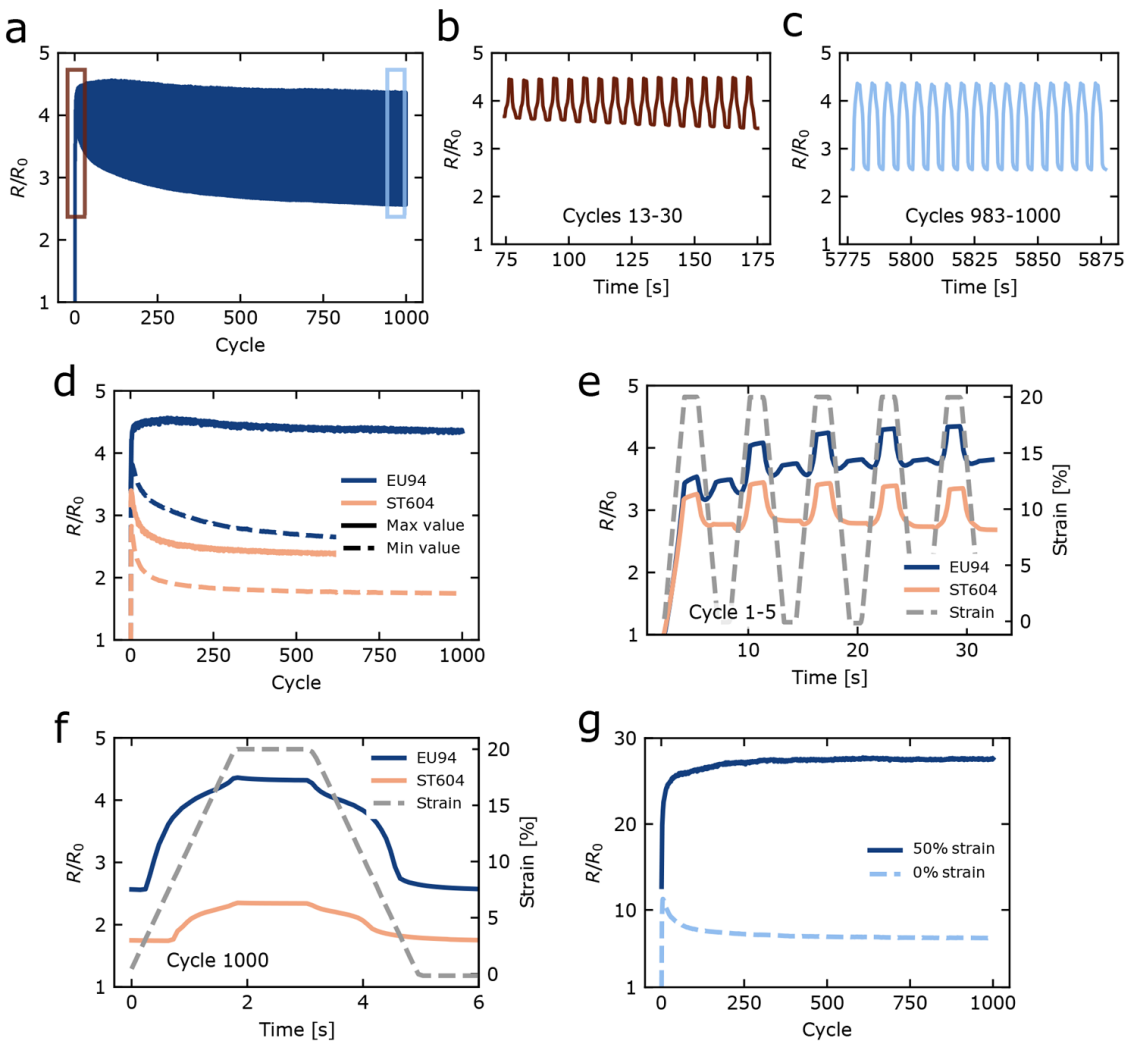
Response of conductors printed on two TPU substrates (EU94 and ST604) submitted to 1000 cycles of 20–50% strain. Time evolution of the resistance normalized over initial resistance *R*/*R*_0_ for a conductive track printed on EU94 for (a) 1000 cycles, (b) cycles 13–30, and (c) cycles 983–1000. (d) Minimum and maximum *R*/*R*_0_ evolving during 1000 cycles for conductors printed on EU94 and ST604. Resistance response to strain during (e) cycles 1–5 or (f) cycle 1000 on EU94 and ST604. (g) Response of a conductor printed on EU94 to 1000 cycles of 50% peak strain.

In these tests, 1 mm wide printed straight lines were subjected to 1000 strain cycles with a cycling rate of 500 mm min^–1^, during which their electrical resistance was monitored over time (Figure 3). Panels a and b of Figure 3 show the resistance during cycling normalized over the resistance before the first strain cycle *R*/*R*_0_ at minimum and maximum strains for printed conductors on the EU94 and ST604 TPU substrates. After an initial peak, the resistance at minimum and maximum strain decreased over time until reaching a plateau. To assess the fatigue behavior in response to repeated stretching, the evolution of the gauge factor GF was monitored during cycling. The gauge factor is a means of quantifying the increase in resistance in response to strain, as defined in eq 1:^49,59,60^
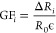
1where *ΔR*_*i*_ = *R*_max,*i*_ – *R*_0_, with *R*_max,*i*_ being the resistance at the maximum strain level of loading cycle *i*, *R*_0_ the initial resistance, and ϵ the peak tensile strain. As shown in Table S4 (section 11.4 of the Supporting Information) and Table 2, the gauge factors are observed to increase during the first few cycles, after which they decay to a stable value of 21 (EU94) or 7.8 (ST604) within 10–200 cycles. The stable behavior of the conductors after the first few cycles as observed in panels a through c of Figure 3 and expressed in the gauge factors will benefit the design of electrical circuits, which are generally designed around a limiting resistance value.^61,62^ Considering that stable values are reached after only 10 strain cycles (EU94) and ∼200 cycles (ST604), prestraining would be a feasible option when manufacturing devices requiring predictable resistance values. In this work, the focus was on a fatigue study of GF. For studying the dynamic behavior of the resistance during cycling, the dynamic gauge factor (DGF) would be more appropriate.^45^ Here, , where *ΔR*_*i*_ = *R*_max,*i*_ – *R*_min,*i*_ and ϵ_*i*_ is the effective strain during cycle *i*. An analysis of the DGF is included in section 11.4 of the Supporting Information. In Table 2 and Table S5, it is shown that the DGF in cycle 1000 assumes very low values of 4.1 and 2.8 for EU94 and ST604, respectively.

**Table 2:** Gauge Factors (GF_*i*_) for Cycles 1 and 1000 and Dynamic Gauge Factors (DGF_*i*_) in Cycle 1000 for Printed Conductors on EU94 and ST604 Substrates Subjected to Peak Strains (ϵ) of 20% or 50%

substrate	ϵ (%)	GF_1_	GF_1000_	DGF_1000_
EU94	20	15.2	20.6	4.1
ST604	20	12.3	7.8	2.8
EU94	50	23.9	54.3	7.6

Several authors have attempted to explain the loading and unloading processes of stretchable conductors composed of metal- or carbon-type particles in a stretchable polymeric matrix.^40,41,63−65^ Generally, the resistance tends to increase during the loading phase, which is termed the positive strain effect.^66^ This increase in resistance is likely induced by rearrangement of junctions between conductive fillers due to macroscopic rearrangements of the TPU-filler network.^40,41,64^ These junctions may be classified into three categories:^40,64^ (i) full contact without contact resistance, (ii) tunneling junction with a distance of <3 nm, and (iii) complete disconnection (>3 nm) due to microcrack formation.

We assume full contact between GNPs with limited contact resistance prior to straining, considering the large flake sizes and surface morphology visible in a scanning electron microscopy (SEM) micrograph of the conductor surface (Figure S15 in section 11 of the Supporting Information), and the relatively high conductivity values of our printed conductors. During the first loading cycle, the resistance of our printed tracks increases considerably (Figure 3d,e and Figure S13), which may, thus, be attributed to the introduction of tunneling junctions and microcracks. The initial jump in peak resistance is followed by a subsequent decrease, which has been observed by other carbon-based^41,45^ and silver-based^35^ conductors, but has remained largely unexplained. Considering that the initial resistance is not recovered after the first strain cycle, we attribute this increase in resistance to the irreversible separation of some interplatelet contacts. As the increase in resistance during the first loading cycle is a factor of only 4, which is low compared to those of most of the works discussed above,^35,41,63^ we hypothesize that the TPU-GNP network is deformed, thus preventing more severe crack formation and propagation and hence the irreversible separation of interplatelet contacts. According to Liu and colleagues, who produced composites from TPU with very low levels of GNP (<0.6 wt %) for strain sensing applications, the rearrangement of the network structure takes a few strain cycles to reach an equilibrium.^41^ We indeed observe a gradual decrease in peak and valley resistances after the first few cycles until a stable level is reached. This indicates that no new cracks are formed during subsequent cycles, while the opening of cracks and breaking of contacts is partially reversible. This conclusion is supported by SEM micrographs of GNP-based conductors at zero strain that have been submitted to cyclic straining (Figure S15), which do not reveal any traces of visible gaps after 1000 strain cycles.

In panels e and f of Figure 3, resistances during individual loading–unloading cycles are presented. During the first loading cycle, the resistance responds linearly to the strain for both substrates (Figure 3e and Figure S13). In contrast, the unloading behavior is nonlinear with the presence of secondary peaks during the unloaded state, in particular for EU94. The shape of the curves changes gradually over time (Figure S14). The magnitudes of the secondary peaks shrink already during the first five cycles until they disappear. Comparable peaks have been observed for other carbon-based conductors^41,67^ but remain largely unexplained. We hypothesize that these features result from the gradual remodeling of the conductive elastic network as discussed above, but this phenomenon requires additional investigation. During later cycles, the resistance responds nonlinearly to the strain during both loading and unloading, as expressed by the presence of peak shoulders, with a gauge factor that appears to be higher during the first 15% than during the remaining 5% of strain. Considering the network model discussed above, this might be attributed to a majority of interplatelet contacts being reversibly disconnected during the first 15% of strain, after which the remaining contacts are more stable. In addition, the substrate appears to be involved in this effect. As detailed in section 11.3 of the Supporting Information, EU94 and ST604 have residual strain levels of approximately 3% and 8%, respectively (Figure S16). Hysteresis is a common phenomenon among thermoplastic polyurethanes.^68^ As a consequence of this hysteresis, the conductor is not actually extended during the first part of the strain cycle. In the first phase of the loading cycle, the slope of the resistance increase is significantly stronger. The shapes of our loading curves, with different gauge factors for the first and second halves of the loading cycle, and the stress–strain curves in Figure S16 display a similar behavior as observed for other TPU-based composites, such as gold films on a TPU substrate,^63^ and GNP-TPU composites with very low GNP loading.^41^ In addition, the (electro)mechanical response of TPU-based composites is generally considered dependent on strain rate, with higher strain rates generally increasing both GF and DGF.^41,68,69^ Considering the high strain rates applied in this study, the presented *R*/*R*_0_ values and (dynamic) gauge factors may therefore be considered an upper bound to the resistance in response to strain exposure. This point is discussed in more detail in section 11.3 of the Supporting Information.

The observed gauge factors are highly robust to fatigue as compared to those of other materials.^35,52,65,70−73^ Traditionally, many printed conductors are manufactured from silver flakes,^2^ but most versions are not stretchable. A few stretchable silver flake-based conductors have been produced through (screen) printing or otherwise.^35,65,70,71^ Although our GNP-based conductors possess higher resistances, they offer superior cyclic stability. Mohammed and co-authors prepared a screen printing ink composed of silver flakes and two polymeric binders.^35^ They subjected the resulting conductors to at least 750 cycles of 20% strain. Although the conductors behaved quite reliably up to 500 cycles with an increase in resistance of approximately 20 times the original value, the resistance was reported to increase unpredictably beyond 750 strain cycles. In other studies in which conductors based on silver flakes were subjected to cyclic strains of 10–50%, the resistance was observed to steadily increase or even shoot up.^65,70,71^ Therefore, serpentine- or meander-shaped printed tracks are often employed to mitigate the effects of straining on metal-based conductors.^4,33−35,52,72,74^ However, such shapes are not desired due to being optimized for only one strain direction.^35,74^ In our case, the gauge factors remain stable during all 1000 strain cycles,and in fact even improve over time. This is a demonstration of high fatigue resistance. The ratio between the resistance after 1000 strain cycles and 300 s of relaxation versus the initial resistance *R*_f_/*R*_0_ is 2.6 for EU94 and only 1.7 for ST604 (Table 3). Unlike the metal-based inks discussed above, repeated straining does not lead to failure, which would prevent application of the tracks as conductors. We ascribe this difference in durability to the high mechanical strength and abrasion resistance of graphene colloids relative to silver flakes. Their high fatigue resistance and low gauge factors make serpentine-shaped tracks obsolete for our GNP-based conductors, with an example of a device shown in section 13 of the Supporting Information (Figure S19). Nonetheless, serpentine-shaped tracks could mitigate the increase in the resistance of the conductors by a factor of almost 2. Further details are provided in section 10 of the Supporting Information. We even subjected a 1 mm straight track on EU94 to a cyclic test with 50% peak strain. Even at those high strain levels, the conductor showed excellent cyclic durability (Figure 3g), as indicated by only very slight changes in the (dynamic) gauge factor after cycle 100 (Table S6 and section 11.4 of the Supporting Information).

**Table 3:** Reduction in Resistance (*R*_0_/*R*_0,p_) Due to Photonic Annealing Relative to Pristine Resistance Values*R*_0,p_ in Table 1 and (dynamic) Gauge Factors (*D*) GF_*i*_ for Cycles 1 and 1000 during Cyclic Straining with Peak Strains of 20% and a Strain Rate of 500 mm min^–1^ of Printed Conductors on EU94 and ST604 Substrates before Post-treatment and after Photonic Annealing with Different Energy Levels Indicated by Their Energy*E* and Voltage*V*^a^

substrate	*E* (J cm^–2^)	*V* (V)	*R*_0_/*R*_0,p_	GF_1_	GF_1000_	DGF_1000_	*R*_f_/*R*_0_
EU94	–	–	1.0	15.2	20.6	4.1	2.6
EU94	0.90	194	0.81 ± 0.02	11.0	20.3	3.0	3.0
EU94	1.40	222	0.31 ± 0.03	7.4	22.2	1.7	3.8
EU94	2.25	260	0.26 ± 0.04	10.1	25.7	1.1	4.3
ST604	–	–	1.0	12.3	7.8	2.8	1.7
ST604	0.62	173	0.81 ± 0.05	17.8	11.2	2.0	2.5
ST604	0.95	198	0.25 ± 0.09	9.5	17.1	1.6	3.2
ST604	1.56	230	0.16 ± 0.04	9.6	18.2	0.7	4.0

a Pulse lengths were fixed at 3 ms. *R*_f_/*R*_0_ expresses the ratio between the resistance after 1000 strain cycles followed by relaxation for 300 s vs *R*_0_. *N* = 4 for *R*_0_/*R*_0,p_, and *N* = 1 for gauge factors and *R*_f_/*R*_0_. Errors represent standard deviations.

In summary, printed stretchable GNP-based conductors have been presented that feature skin-compatible materials, high conductivity, stretchability up to at least 100% strain, low gauge factors, and excellent fatigue resistance at elongations of 20% and 50%. This makes these conductors ideal for use in wearable electronics and does not require serpentine-shaped designs. In the following section, we will describe how cyclic durability and conductivity can be further optimized and tuned using photonic annealing.

### Effect of Photonic Annealing on the Electromechanical Behavior of Printed Conductors

Commonly, thermal annealing is applied to improve the conductivity of graphene-based conductors. However, the high temperatures negatively impact the stretchability of flexible substrates.^75,76^ Alternatively, photonic annealing^55,75,77^ and compression rolling^75^ can drastically reduce the resistance of printed graphene-based conductors without affecting the substrate. In photonic annealing with intense pulsed light (IPL), intense bursts of broad-spectrum light with wavelengths between 200 and 1500 nm are emitted from a xenon flash lamp.^78−80^ As the GNPs are highly light absorbing in contrast to the substrate and much more conductive to heat than the substrate, the absorbed light causes fast heating of only the ink layer. Because the high temperatures required for thermal degradation of the binder are reached for a very short time on a scale of milliseconds, the substrate remains unaffected,^55,78,81^ and stretchability of the substrate is preserved.^76^ Yang et al. demonstrated that photonic annealing with different energy intensities could be used to modulate the electrical response of stretchable silver-based conductors to repeated strain.^76^ Increasing photonic annealing energy inputs reduced both the initial resistance and the (dynamic) gauge factor. We hypothesized that this principle could also be applied to modulate the electromechanical response of our GNP-based conductors. Therefore, the impact of several levels of photonic annealing on the sheet resistance and gauge factor was studied.

Each combination of ink and substrate generally requires different photonic annealing conditions.^55,81^ As both of our TPU substrates have different degrees of transparency, this was expected to hold true even for the two TPU materials. The differences in thermal absorption were confirmed by transmission measurements using a built-in bolometer, where identical photonic annealing conditions revealed a transmitted energy of 35% for EU94 or 76% for ST604. Therefore, the photonic annealing process was optimized independently for the two substrates to minimize the sheet resistance without delamination, but conditions were maintained as similar as possible for comparability between the two substrates.

**Figure 4:**
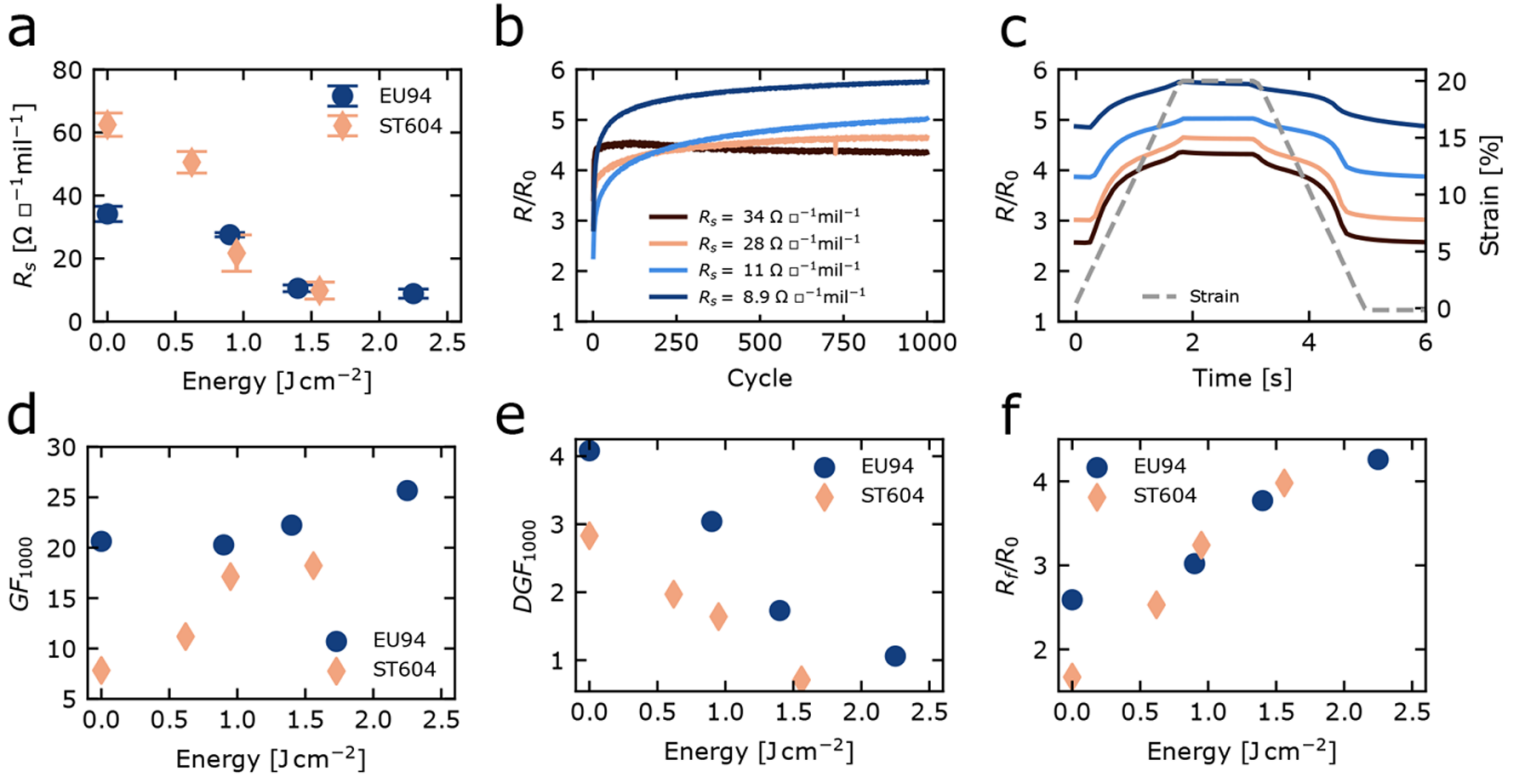
Effect of photonic annealing with increasing energy levels on the sheet resistance and electromechanical response to repeated stretching with a peak strain of 20%. (a) Sheet resistance vs photonic annealing energy (*N* = 4). Development of peak resistance *R*/*R*_0_ for conductors printed on EU94 exposed to different photonic annealing energies *E* of 0.90, 1.4, and 2.3 J cm^–2^ (b) over 1000 cycles and (c) during cycle 1000. (d) GF_1000_ vs photonic annealing energy. (e) DGF_1000_ vs photonic annealing energy. (f) Recovery of the initial resistance after 1000 strain cycles followed by relaxation for 300 s (*R*_f_/*R*_0_) for different photonic annealing energies. *R*_s_ values represent the averages derived from Table 1 multiplied by the *R*_0_/*R*_0,p_ factor in Table 3.

In an initial experiment, the pulse duration, voltage, and consequently energy density were varied. The differences between different pulse lengths on the achieved resistance values turned out to be minimal. Therefore, we opted for an intermediate pulse length of 3 ms. For each substrate, the administered power density was optimized for this pulse length by increasing the voltage until a minimum resistance was achieved. This combination of the voltage and pulse length was taken as the maximum energy level of the series. Additionally, two energy levels of ∼60% and ∼40% of the highest energy were added by selecting lower voltages (Table 3) to investigate the tunability of the response of the conductors to cyclic strain. As shown in Table 3, photonic annealing gradually reduced the resistances of printed tracks down to values of only 26% (EU94) or 16% (ST604) of their value before postprocessing, resulting in *R*_s_ values of ≤10 Ω □^–1^ mil^–1^ (Figure 4a). This reduction in resistance is comparable to previous results on 6 μm thick graphene layers stencil-printed on flexible substrates.^75^

To evaluate whether stretchability was maintained after photonic annealing, the post-treated conductors were submitted to a cyclic strain test consisting of 1000 cycles with a 20% peak strain. Figure 4b shows the evolution of the normalized peak resistance (*R*/*R*_0_) for conductors on EU94 post-treated with various energy levels. Excitingly, the stretchability and fatigue resistance of the conductors are preserved after photonic annealing at all energy levels, which is indicated by the flat curves with *R*/*R*_0_ values only slightly higher than for the pristine conductor. Despite a significant reduction in sheet resistance by a factor of 4, the gauge factors in the final cycle (GF_1000_) of the conductors deviate only slightly from the pristine value (Figure 4d and Table 3). In contrast, the dynamic gauge factors (DGF_1000_) decreased drastically with greater energy input to remarkably low values (Figure 4e and Table 3). The latter is also illustrated in Figure 4c, which shows gradual flattening of the resistance curves in cycle 1000 for conductors on both substrates. While DGF_1000_ decayed with an increase in energy input, the recovery of the initial resistance after straining followed by relaxation for 300 s is reduced, resulting in higher *R*_f_/*R*_0_ ratios (Figure 4f). Photonic annealing did not affect the residual strain or the elastic moduli of the TPU substrates (Table S7 and section 11.5 of the Supporting Information), which confirms the minimal effect of the energy burst on the substrate.

Our results are in line with observations of photonic annealing on silver nanowire-based stretchable conductors, where increased energy inputs also reduced the initial resistance values and the dynamic gauge factors.^76^ Unlike these authors, we did not observe any flattening of the curves for *R*/*R*_0_ versus cycle number, because our pristine conductors already demonstrated very stable resistance–strain behavior during cycling. In their article, the authors attribute the improved performance to an increased number of interconnections between nanowires. We believe a loss of flexibility of the TPU-GNP network due to carbonization of the TPU binder might reduce the elasticity of the composite, resulting in a reduced dynamic gauge factor while simultaneously complicating recovery of the resistance after use, resulting in an increased *R*_f_/*R*_0_. Nonetheless, the postprocessed tracks remain highly suitable for application as stretchable conductors as indicated by the preserved stretchability and fairly stable gauge factors in Figure 4, with an example of a device shown in section 13 of the Supporting Information (Figure S19). The gauge factors remained even more stable after the first few cycles for conductors printed on ST604 than on EU94. Otherwise, trends for ST604 were similar. The ST604 resistance–strain curves are presented in the Supporting Information (section 12 and Figure S18).

Duplicates of the entire annealed series were also compressed to study the additional effects of compression rolling on strain behavior. Generally, compression rolling is used to restore the structural integrity that is partially lost during photonic annealing due to binder degradation.^75^ However, compression did not improve the resistance values and had a limited impact on stretchability, which is shown in the Supporting Information (section 12 and Table S8). We believe this is due to the stretchable substrate absorbing part of the compressive stresses or due to the rollers extending the substrate during compression, essentially creating a prestrained track.

## Conclusions

We formulated a TPU- and GNP-based ink for screen printing of nontoxic stretchable conductors on several stretchable and flexible substrates with feature sizes down to 200 μm. The ink yields highly conductive, stretchable tracks of only 34 Ω □^–1^ mil^–1^ on TPU before any postprocessing that remain conductive even at 100% strain. The printed conductors were submitted to 1000 strain cycles of 20% and 50% peak strain. Unlike most silver conductors, the GNP-based conductors proved to be strongly resistant to fatigue and exhibit low gauge factors. We demonstrated that photonic annealing may be used to modulate the sheet resistance of GNP-based stretchable conductors to remarkably low levels while preserving stretchability and tuning drift. The tunability of the ink rheology offers perspective for printing with large-volume roll-to-roll technologies beyond screen printing, such as flexographic printing, as demonstrated already, and might be extended to other 2D material ink formulations using similar solvent-exchange approaches as shown here. The flexibility and scalability of the ink formulation and postprocessing open a route toward the industrial production of flexible and stretchable conductors for wearable electronics applications.

## Experimental Section

### Preparation of Intercalated and Thermally Expanded Graphite

The −10 mesh natural graphite (Alfa Aesar) was intercalated with sulfuric acid (95–97%, Sigma-Aldrich) and potassium permanganate (Sigma-Aldrich) as previously described.^20^ The resulting intercalated graphite was left to dry for at least 3 days and subsequently thermally expanded in a home appliance microwave oven (LG Smart Inverter Magnetron) for 5 min at 1100 W.

### Preparation of Graphene Preink by High-Shear Mixing

In a typical synthesis, 0.5 g of ethyl cellulose (22 cP, Aldrich Chemistry) was dispersed in 400 mL of ethyl acetate (Biosolve Chimie) and 100 mL of isopropyl alcohol (VWR Chemicals) by mixing for 5 min at 7000 rpm with an Ystral X40/38 high-shear mixer equipped with a stator with an internal diameter of 35 mm and a 25 mm rotor. A graphene nanoplatelet dispersion was produced by adding 5 g of thermally expanded graphite and mixing for 1 h at 7000 rpm. A GNP:TPU binder mass ratio of 1:3 was selected as a trade-off between printability and conductivity. To achieve this ratio, 45 g of Neorez U-431 binder (Covestro) was incorporated, followed by mixing at 5000 rpm for 5 min, adding 110 mL of propylene glycol ethers (Dowanol PnB, Sigma-Aldrich), and mixing for an additional 10 min. During high-shear mixing, the dispersion was cooled with ice–water.

### Solvent Exchange and Gelation of Graphene Dispersion

The entire preink volume was transferred to a round-bottom flask for solvent exchange at a Hei-VAP precision rotary evaporator (Heidolph Instruments GmbH). The solvent was evaporated at 73 °C at decreasing pressure, until no more distillate was collected after 1 h at 200 mbar. The thick, homogeneous residue was used as ink without any further treatment. The produced ink volume was around 100–150 mL. The GNP loading was 4 wt %.

### Rheology

The rheological behavior of each ink was characterized at 20 °C with duplicate measurements on an Anton Paar Physica MCR301 rheometer equipped with a parallel plate measurement system with a diameter of 25 mm. After the application of ink between the plates, the gap was set to 1 mm and the shear rate was gradually increased from 0.001 to 1000 s^–1^ while the shear viscosity was recorded at 31 intervals, for 20 s each. Peak-hold tests or three-interval thixotropy (3ITT) tests were performed in rotary mode, measuring for 60 s at a γ̇ of 0.1 s^–1^, followed by 60 s at a γ̇ of 100 s^–1^ and 120 s at a γ̇ of 0.1 s^–1^. A measurement was performed every 4 s.

### Printing and Post-treatment

Inks were blade coated on glass substrates with an Erichsen Quadruple Film Applicator (model 360) with a gap height of 120 μm and a width of 13 mm. The length of printed tracks was approximately 55 mm. Screen printing was carried out with a DEK Horizon 03i (DEK International) semiautomatic screen printer with a 45° polyurethane squeegee with a print speed of 80 mm s^–1^ and a print gap of 1.6 mm. We employed a 200 mesh metal mesh screen with a 12 μm thick emulsion layer and a theoretical wet layer thickness of 43–55 μm (KOENEN GmbH, Ottobrunn-Riemerling). Structures were printed on PET (MELINEX ST504, DuPont Teijin Films) and two types of TPU substrates, EU94 (DelStar Technologies) and ST604 (Bemis Associates), with root-mean-square (RMS) roughness values (section 5 of the Supporting Information) of 2.0, 2.1, and 6.5 μm, respectively, and surface energies as determined with Dyne inks (JARP) of approximately 42, 34, and 38 mN/m, respectively. Prints were cured for 15 min at 120 °C. A subset of printed conductors were post-treated with a PulseForge 1200 Photonic Curing System (PulseForge) for photonic annealing with IPL. The pulse length was fixed at 3 ms, while the voltage was varied as specified in Table 3. After photonic annealing, some samples were compressed between 0.25 mm polycarbonate sheets to prevent direct contact of the printed graphene and rollers with a HBM compression rolling system (HBM Machines).

### Electrical, Morphological, and Mechanical Characterization

The conductor profile of blade-coated and screen-printed samples was measured with a Dektak Bruker XT profilometer with the following settings: standard scan, probe height of 524 μm, hills and valleys, stylus radius of 2 μm, resolution of 0.833 μm/point, and force of 3 mg. Because the profiles showed large variations in height, the baseline thickness was derived as an indication of the conductive path according to a procedure detailed in the Supporting Information (section 5 and Figure S7). For the screen-printed thin lines, 10 samples were profiled with two lines of 1 mm (2400 data points) per sample.

Electromechanical characterization was performed with a Mark-10 straining apparatus (model ESM303) equipped with a Keithley 2612A System SourceMeter (four wires) and a Leica Z16 APO microscope (Figure S10). For the strain test with an increasing strain amplitude, samples (width of 22 mm, length of 102 mm) with printed tracks (width of 1 mm, length of 76 mm) were strained at 200 mm min^–1^ for 50 cycles with peak strains of 2–100% and a linear increase in peak strain of 2% per cycle. For the cyclic straining tests, samples were strained 1000 times with a peak strain of 20% and a loading and unloading rate of 500 mm min^–1^. In all tests, waiting times of 1 s were maintained at the maximum and minimum strain levels. Gauge factors during cycling were calculated according to eq 1. Sheet resistance values were obtained by dividing the line resistance obtained with the Keithley 2612A System SourceMeter (four-wire measurement) by the number of squares between the electrodes,^82^ followed by averaging over 10 samples per substrate. Data analysis, statistical tests, and data visualization were performed with R and Python.

## Supporting Information Available

Figures S1–S19 and Tables S1–S8 containing scanning electron micrographs of raw graphite, intercalated graphite, thermally expanded graphite, and graphene nanoplatelets (GNPs), size distributions of GNPs obtained with dynamic light scattering, oscillatory rheology of the inks, sheet resistance values for blade-coated conductors on glass, pictures of screen prints on flexible substrates, conductor profiles, baseline thicknesses and details about the determination of the baseline thickness and the abrasion resistance of the conductors, a scan of a flexographic print, details about the setup for electromechanical characterization, optical micrographs of samples before, during, and after straining, gauge factors for the straining tests with increasing strain amplitude, a comparison between the electromechanical response of straight lines versus serpentine structures, resistance–strain plots, normalized resistance during selected cycles of a cyclic strain test, scanning electron micrographs of conductors before and after straining, a discussion of the residual substrate strain, stress–strain curves, (dynamic) gauge factors during cyclic strain tests with 20% and 50% peak strain, elastic moduli, additional data about the photonic annealing experiments, and stretchable wristband with a circuit composed of the stretchable tracks discussed in this paper that connect an LED to a battery.
