# Supplementary material for: Printed Stretchable Graphene Conductors for Wearable Technology
Source: Chem Mater. 2022 Aug 29;34(17):8031–42. doi: 10.1021/acs.chemmater.2c02007 (PMC9477090; doi:10.1021/acs.chemmater.2c02007)
Supplement: Supplementary file 1 — cm2c02007_si_001.pdf [file cm2c02007_si_001.pdf]

Supporting Information

Printed stretchable graphene conductors for wearable technology

Laura S. van Hazendonk,[†] *Artur M. Pinto,*[†,ǂ] *Kirill Arapov,*[†] *Nikhil Pillai,* [⁋] *Michiel R.C. Beurskens,*[†] *Jean-Pierre Teunissen,*[§] *Asko Sneck,* [‖] *Maria Smolander,* [‖] *Corne H.A. Rentrop,*[§] *Piet C.P. Bouten,*[§] *and Heiner Friedrich*[*,†,⊥]

† Laboratory of Physical Chemistry & Center for Multiscale Electron Microscopy, Department of Chemical Engineering and Chemistry, Eindhoven University of Technology, PO box 513, 5600MB, Eindhoven, The Netherlands
ǂ LEPABE, Faculdade de Engenharia, Universidade do Porto, 4200-180, Porto, Portugal
⁋ PulseForge, 400 Parker Dr, Suite 1110, 78728, Austin TX, USA
§ Holst Centre – TNO, High Tech Campus 31, 5656 AE, Eindhoven, The Netherlands
‖ VTT Technical Research Centre of Finland Ltd., PO Box 1000, FI-02044 VTT, Espoo, Finland
⊥ Institute for Complex Molecular Systems, Department of Chemical Engineering and Chemistry, Eindhoven University of Technology, PO box 513, 5600MB, Eindhoven, The Netherlands

Email: h.friedrich@tue.nl

Contents

1. Graphite exfoliation into graphene nanoplatelets 3
2. Rheology of GNP-based inks 5
3. Blade coating on glass 6
4. Screen printing on flexible substrates 7
5. Conductor profiles and determination of baseline thickness 8
6. Abrasion resistance of screen-printed conductors 10
7. Flexographic printing 11
8. Setup for electromechanical characterization 12
9. Strain test with increasing strain amplitude 13
10. Serpentines vs straight lines: Electromechanical response 14
11. Repetitive strain tests with 20-50% peak strain 15
11.1 Resistance response 15
11.2 Scanning electron micrographs 16
11.3 Residual strain of substrates due to rapid cycling 17
11.4 Gauge factors 19
11.5 Elastic moduli 20
12 Photonic annealing 21
13 Wristband 23
14 References 24

1. Graphite exfoliation into graphene nanoplatelets

As is described in the experimental section of the main text, raw graphite was intercalated with sulfuric acid and potassium permanganate and subsequently thermally expanded in a microwave before exfoliation into graphene nanoplatelets (GNPs).[1,2] This allowed high exfoliation at relatively low shear rates in order to maintain rather large platelets, resulting in highly conductive printed tracks. Figure S1 shows morphological changes induced to raw graphite by intercalation and thermal expansion. Figure S2 presents GNP size distributions obtained via dynamic light scattering (DLS) after removal of the largest platelets via 1h settling. The DLS data indicate a polydisperse GNP population with an average hydrodynamic diameter of 2.3 ± 0.4 µm. This polydispersity is corroborated by the micrographs in Figure S3. From the optical micrograph in Figure S3a, it becomes apparent that the sample contains some larger ~50 µm particles and many smaller flakes on the order of a micrometer (pixel resolution: 212 nm/pixel). From the thickness contrast, it can be concluded that we obtained multilayer graphene nanoplatelets that most likely will behave as stiff plates.[3] With scanning electron microscopy, the multilayer nature of the larger flakes could be resolved (Figure S3b-d).

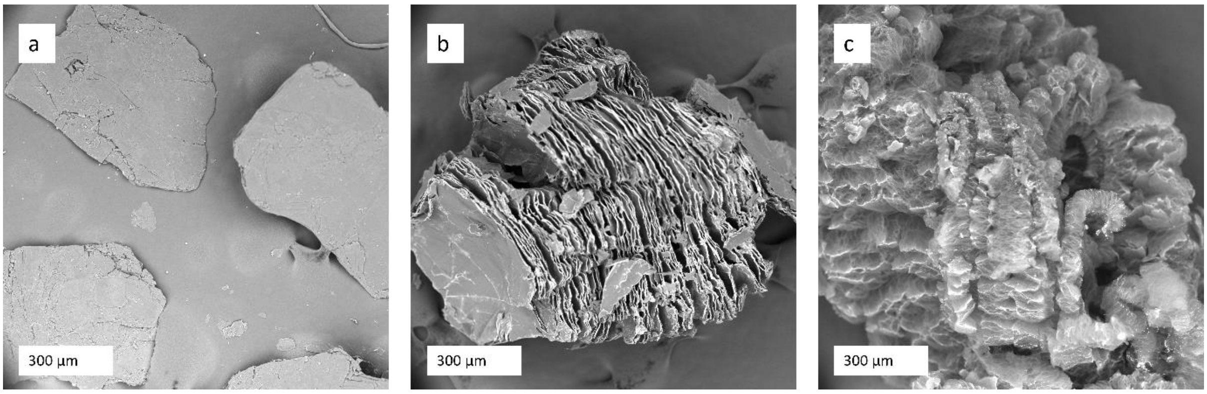

Figure S1. Scanning electron micrographs of a) raw graphite; b) intercalated graphite and c) thermally expanded graphite. Scanning electron micrographs were obtained with secondary electron contrast at 10 kV and 40.3 µA on a Phenom ProX SEM (Thermo Fisher Scientific). Magnifications were 220x.

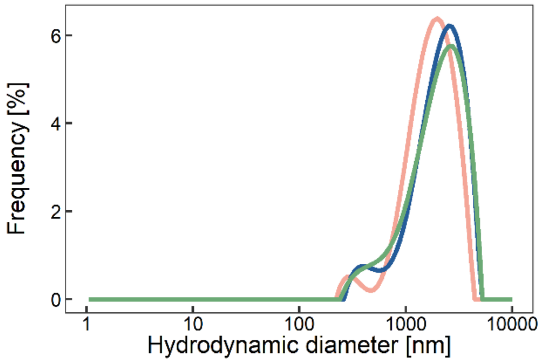

Figure S2. Intensity-weighted size distribution of a typical dispersion containing graphene nanoplatelets (GNPs) and ethyl cellulose (EC) in ethyl acetate and ethanol (4:1) characterized with dynamic light scattering (DLS) on an Anton Paar LiteSizer after removal of the largest platelets via 1h sedimentation. The measurement was performed in triplicate, which yielded a hydrodynamic diameter of 2.3 ± 0.4 µm.

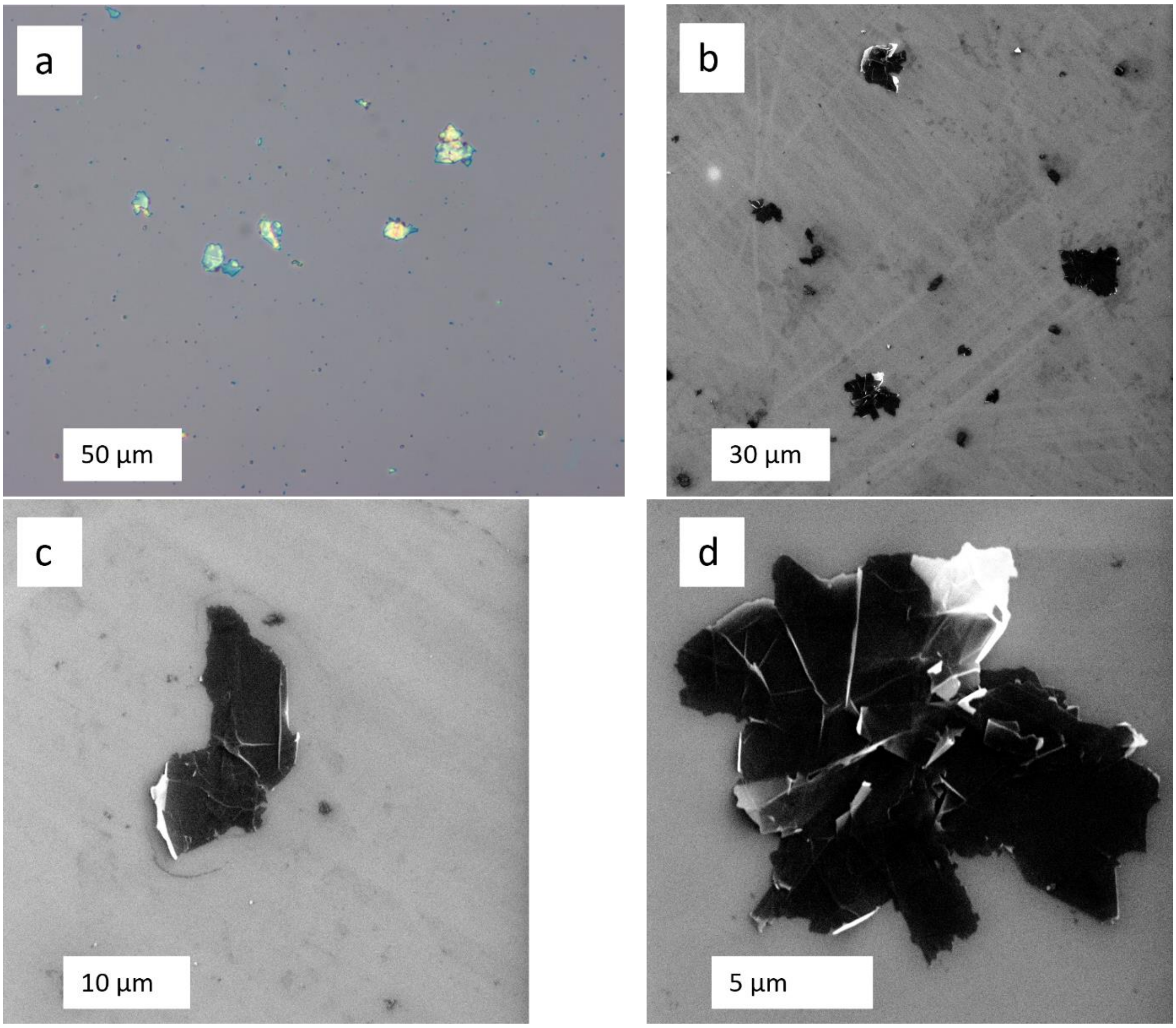

Figure S3. Micrographs of a typical dispersion containing graphene nanoplatelets (GNPs) and ethyl cellulose (EC) in ethyl acetate and ethanol (4:1) drop casted on a silica wafer; a) optical micrograph acquired with a Zeiss Axioplan optical microscope equipped with a LD Epiplan 50x objective; b-d) Scanning electron micrographs were obtained with secondary electron contrast at 10 kV and 40.3 µA on a Phenom ProX SEM (Thermo Fisher Scientific). Magnifications were 2000× (b), 6500× (c) and 16500× (d).

2. Rheology of GNP-based inks

In addition to the rotational rheology shown in Figure 1 of the main text, oscillatory rheology was performed to obtain a basic understanding of the viscoelastic behavior of the GNP-TPU inks. First, an amplitude sweep was performed with increasing shear strain γ from 0.01-100% and fixed angular frequency ω = 10 rad/s. From the results in Figure S4a and b, it might be concluded that the storage modulus G' is dominant over the loss modulus G" over almost the entire shear range, which is also apparent from the loss factor tan δ = G"/G' slightly below 1. The clear contributions from both G' and G'' to the ink rheology with a dominant loss modulus imply that the ink behaves like a viscoelastic solid.[4] We hypothesize that this behavior is due to the presence of a jammed platelet network. The loss factor gradually increases with shear strain until it rises more drastically at shear strains beyond 30%, which we attribute to destruction of the ink microstructure.

Next, a frequency sweep was performed by varying the angular frequency ω from 0.1 to 100 rad s^{-1} at fixed shear γ = 0.01% (Figure S4c-d). At this small shear amplitude, both G' and G" and, hence, the loss factor are relatively frequency-independent. This is another indicator of a solid-like jammed network structure, which, at least at these low strain amplitudes, is able to elastically store energy in the form of network-like particle-particle interactions.[4,5]

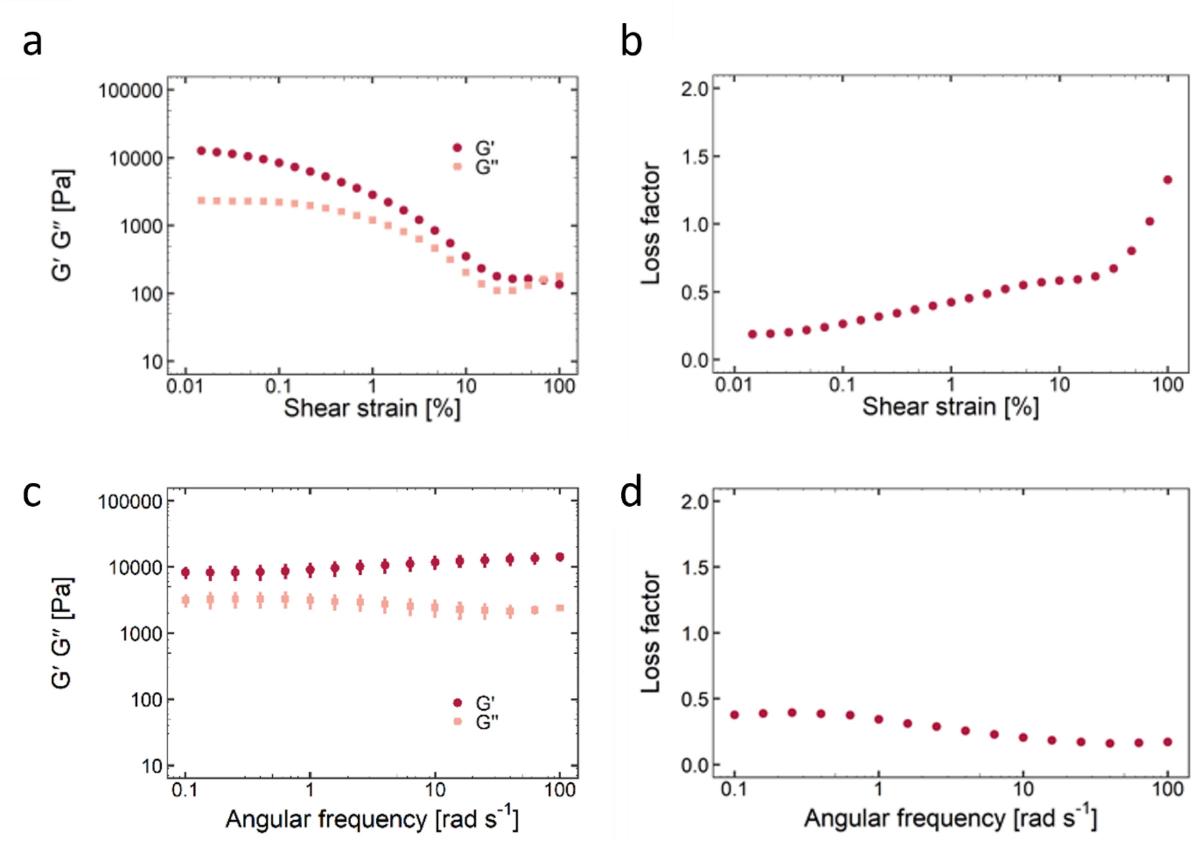

Figure S4. Oscillatory rheology of a typical GNP-TPU ink; a) storage (G') and loss (G") moduli and b) corresponding loss factor tan δ = G"/G' as measured in an amplitude sweep with γ varied from 0.01 to 100% and ω = 10 rad/s; c) storage (G') and loss (G") moduli and d) corresponding loss factor tan δ = G"/G' as measured in a frequency sweep with γ = 0.01% and ω = 0.1 - 100 rad/s. All measurements were performed in duplicate and error bars indicate the standard deviation (some are invisible due to their small magnitude).

3. Blade coating on glass

Table S1. Sheet resistances and baseline thicknesses of five replicate GNP inks blade coated on glass (55 x 12 mm; N = 3). Errors represent the standard deviation.

	R_s a) [Ω □$^{-1}$ mil^{-1}]	Thickness b) [μm]
Ink 1	42 ± 5	12 ± 0.2
Ink 2	30 ± 3	9 ± 0.6
Ink 3	39 ± 4	9 ± 0.6
Ink 4	36 ± 3	9 ± 0.5
Ink 5	45 ± 3	10 ± 0.4

a) The sheet resistances for these glass samples were obtained with a Keithley 237 High Voltage Measure Unit as source and a Keithley 6517A High Resistance Meter arranged in a linear setup with an interprobe distance of 5 mm. The sheet resistances R_s were calculated according to $R_s = C\frac{\pi}{\ln(2)}R$, with R the resistance. A correction factor C of 0.52 was applied to correct for the coating geometry (55 x 12 mm) deviating from an infinite square.[1] $\bar{R}_s$ = 38 ± 6 Ω □$^{-1}$ mil^{-1}, corresponding to a conductivity S = (1.1 ± 0.2) · 10^3 S m^{-1} and a resistivity ρ = (9.6 ± 1.5) · 10^{-4} Ω m; b) The baseline thickness was obtained according to a procedure detailed in section 3 of the SI based on a line of 12 mm (1.4 ·10^4 data points).

4. Screen printing on flexible substrates

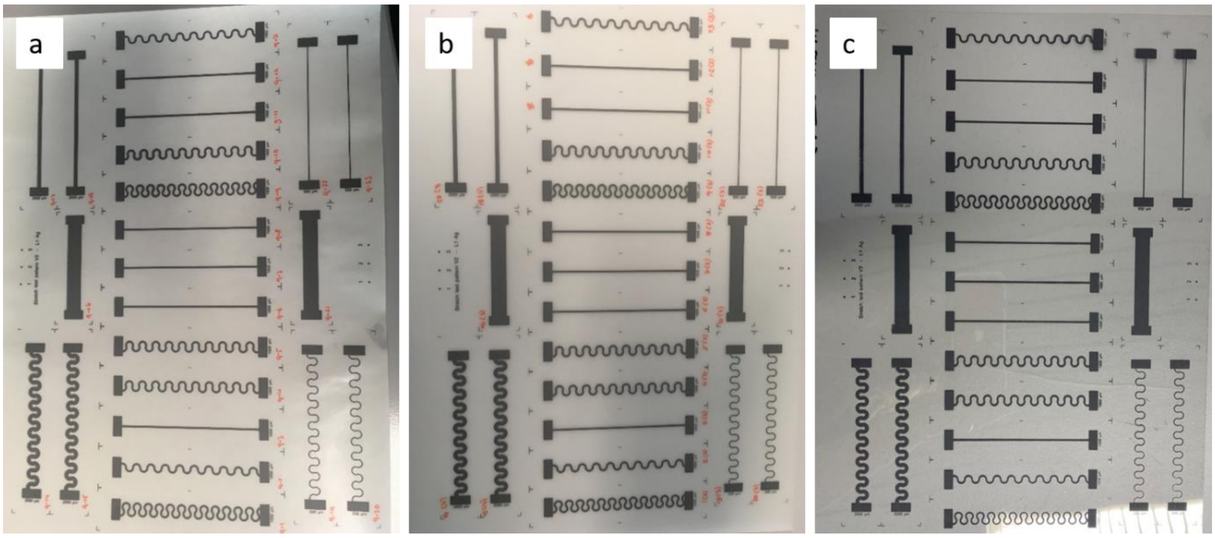

Figure S5. Photograph of the test patterns printed on a) EU94 TPU; b) ST604 TPU and c) ST504 PET with a 200 mesh KOENEN screen with a theoretical wet layer thickness of 43-55 µm.

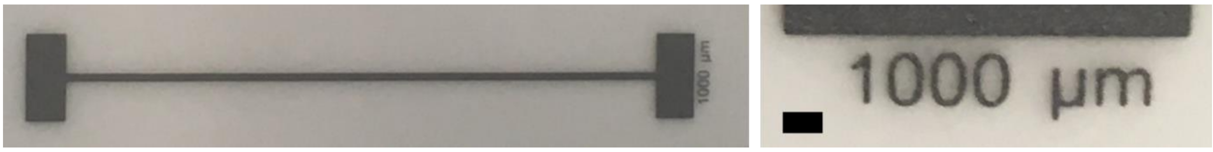

Figure S6. Photograph of a printed track (width: 1 mm, length: 76 mm) used for electromechanical characterization and profilometry; (left) Full structure; (right) zoom with scale bar denoting 1 mm. The smallest feature has a width of 200 µm.

5. Conductor profiles and determination of baseline thickness

Profiles of printed conductors were rather rough (Figure S7). In this work, the baseline thickness (orange continuous lines in Figure S7) was used as an indication of the active thickness of the conductive path, because it corresponds to the thickness of a homogeneous layer that contributes to conductivity. We expect that platelets sticking out do not contribute as much to in-plane conductivity.

The baseline thickness was obtained according to the following procedure: The conductor profile was sampled with a Dektak Bruker XT profilometer with a resolution of 0.833 µm/pt, a stylus radius of 2 µm and a force of 3 mg. The manufacturer's software was used to level the dataset. For each print type, 3, 5 or 10 samples were profiled (Table S2), resulting in $\geq 9 \cdot 10^3$ data points per print type. For the data analysis, peaks and valleys were extracted from the dataset with the findPeaks and findValleys functions from the R package quantmod.[6] Negative datapoints were excluded from the dataset, as they would likely refer to pinholes or mistakes in the levelling process. Next, the mean peak height was calculated for all peaks > 10 µm. Next, the valley data points exceeding the mean peak height were discarded, as they likely refer to shoulder peaks. Finally, the mean baseline thickness was calculated from the remaining valleys. In Figure S7, the baseline, mean and peak thicknesses are plotted for representative samples of each conductor type.

Table S2 provides the mean thickness and baseline thickness for printed conductors on each of the substrates. In both cases, the print layer is thicker on TPU ST604 than on TPU EU94 and PET ST504. This may be related to a difference in substrate roughness. The root-mean-square (RMS) substrate roughness $R_{RMS} = \sqrt{\frac{1}{n}\sum_{i=1}^{n} y_i^2}$[7] with n the number of profilometer data points i, and y_i the height at point i is 2.0 for PET ST504, 2.1 µm for TPU EU94 and 6.5 µm for ST604.

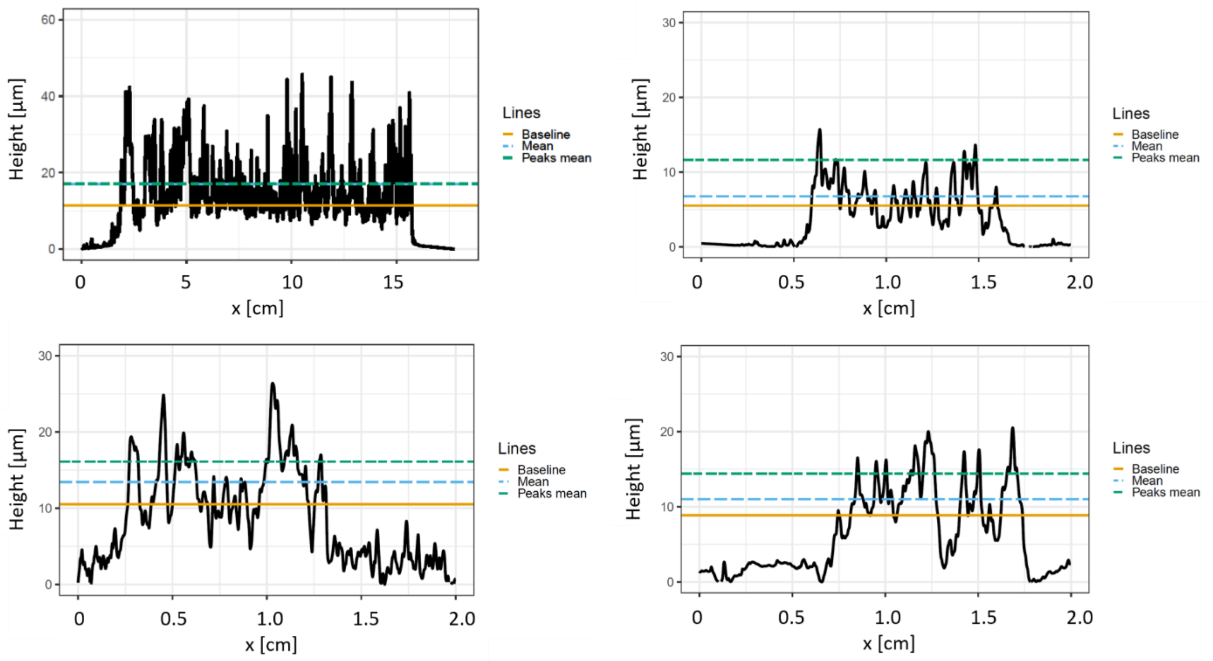

Figure S7. Representative sample profiles obtained with a Dektak Bruker XT profilometer with lines indicating the mean, baseline and peak thicknesses of an ink a) blade coated on glass; b) screen printed on PET ST504; c) screen printed on TPU ST604 and d) screen printed on TPU EU94.

Table S2. Mean and baseline thicknesses as well as (normalized) sheet resistances of tracks of different geometries printed (EU94, ST604, PET) or blade coated (glass) on several types of substrates.

	**Thickness**[a)] **[µm]**	**Baseline**[a)] **[µm]**	$\boldsymbol{R_s}$[b)] **[Ω □$^{-1}$ mil^{-1}]**	**Lines/ sample**[c)]	**Samples**
EU94 0.5x76mm	8.3 ± 0.6	6.3 ± 0.4	41 ± 2.8	3	5
EU94 1x76mm	7.9 ± 1.4	5.8 ± 1.2	34 ± 2.4	2	10
EU94 2x76mm	6.2 ± 0.6	4.5 ± 0.3	45 ± 3.5	2	5
EU94 8x60mm	6.0 ± 0.7	4.5 ± 0.6	48 ± 1.6	1	5
ST604 0.5x76mm	13.7 ± 1.2	10.2 ± 1.1	60 ± 5.9	3	5
ST604 1x76mm	15.4 ± 1.3	11.5 ± 1.0	62 ± 3.7	2	10
ST604 2x76mm	11.4 ± 1.1	8.4 ± 1.0	69 ± 3.1	2	5
ST604 8x60mm	9.2 ± 1.6	6.7 ± 1.4	59 ± 4.8	1	5
PET 0.5x76mm	7.4 ± 0.3	5.4 ± 0.3	31 ± 1.3	3	5
PET 1x76mm	7.8 ± 0.6	5.7 ± 0.5	30 ± 2.6	2	10
PET 2x76mm	6.1 ± 0.4	3.9 ± 0.4	30 ± 2.1	2	5
PET 8x60mm	5.4 ± 0.4	3.7 ± 0.3	24 ± 1.5	1	5
Glass 12x55mm	16.5 ± 0.5	10.5 ± 0.4	11 ± 0.4	1	3

[a)]The (baseline) thicknesses were extracted from the profilometer datasets as described above.
[b)]Resistances were measured with triplicate measurements on a Keithley 2612A System SourceMeter (4-point measurement). From these data, sheet resistances were obtained by dividing the line resistances by the number of squares between the electrodes and by using the baseline thickness to normalize to a layer thickness of 25 µm as described in the experimental section of the main text.
[c)]The number/lines per sample refers to the number of profilometer tracks measured orthogonal to the conductor’s longitudinal axis. This number of lines resulted in $\geq 9 \cdot 10^3$ data points/sample.

6. Abrasion resistance of screen-printed conductors

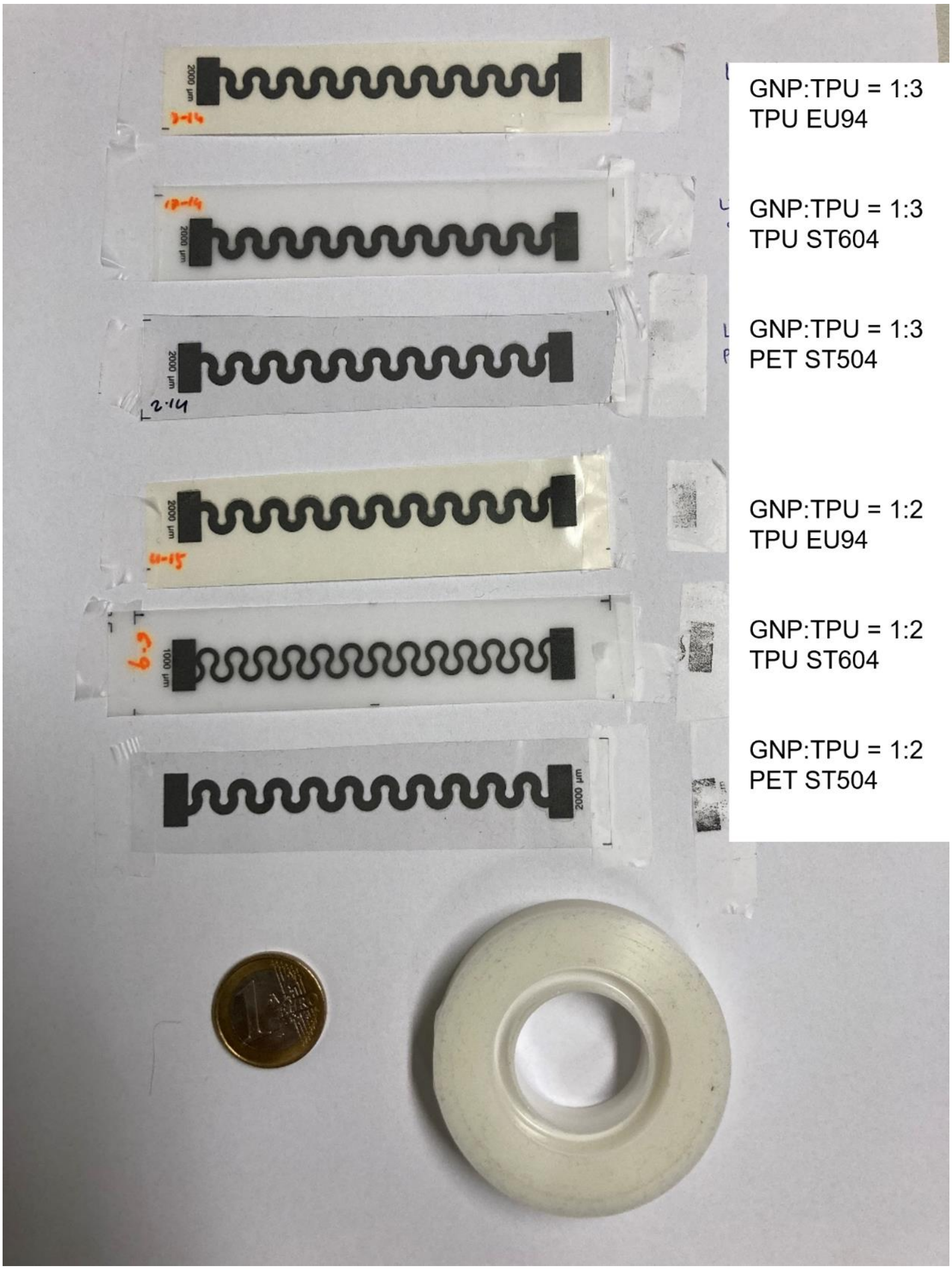

Figure S8. Abrasion resistance of printed conductors formulated with two different GNP:TPU ratios printed on three different substrates. Tape tests were performed with Scotch® magic^{TM} tape (3M), which was placed on the sample with a 1 euro coin (mass = 7.5 g, diameter = 23.25 mm, pressure = 173 N m^{2}) on top for 30 s. Tape samples on the right display the material removed from the printed tracks. The conductors formulated with a GNP:TPU ratio of 1:3 (as used in this work) are more abrasion resistant than the conductors containing GNP:TPU = 1:2.

7. Flexographic printing

A flexographic print test was performed using an ink similar to the one presented in the article. The ink contained 7.6 wt% graphene nanoplatelets (GNP) purchased from Sigma Aldrich (grade M5), 22.8 wt% thermoplastic polyurethane (TPU) binder, 1.1 wt% ethyl cellulose (EC) and 68.4 wt% solvent (dowanol PnB). Printing was performed on paper using an RK Flexiproof 100 flexo printer with a 1.7 mm thick printing plate designed for use with solvent-based inks. As shown in Figure S9, horizontal and vertical lines of various widths are printed fairly successfully on paper for all layer widths.

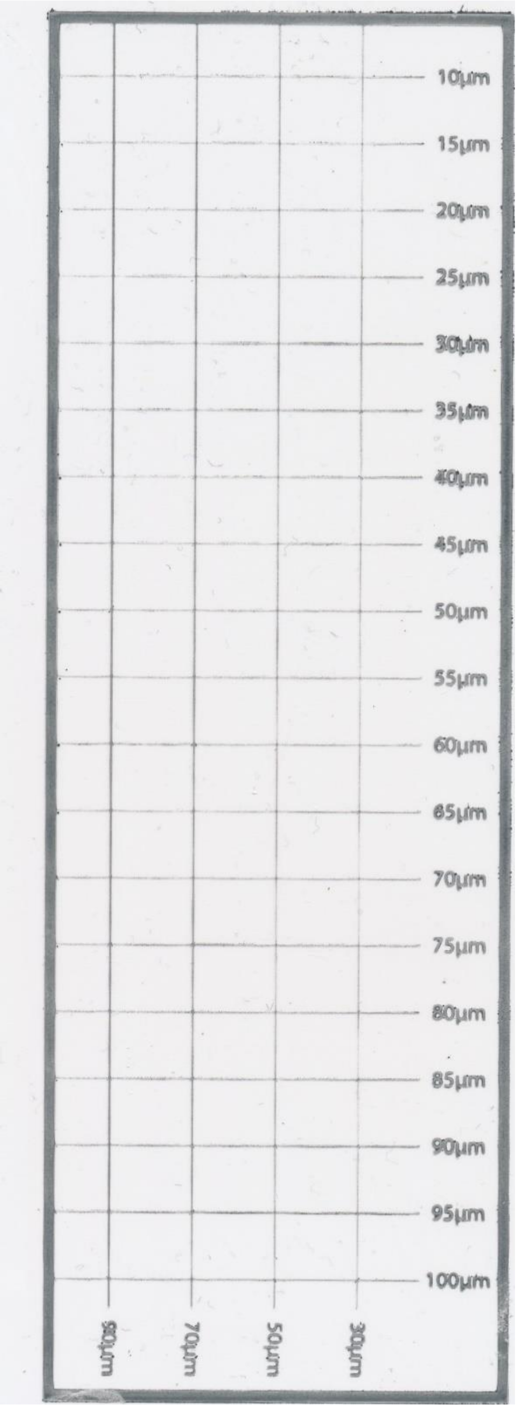

Figure S9. Scan of a flexographic print on paper.

8. Setup for electromechanical characterization

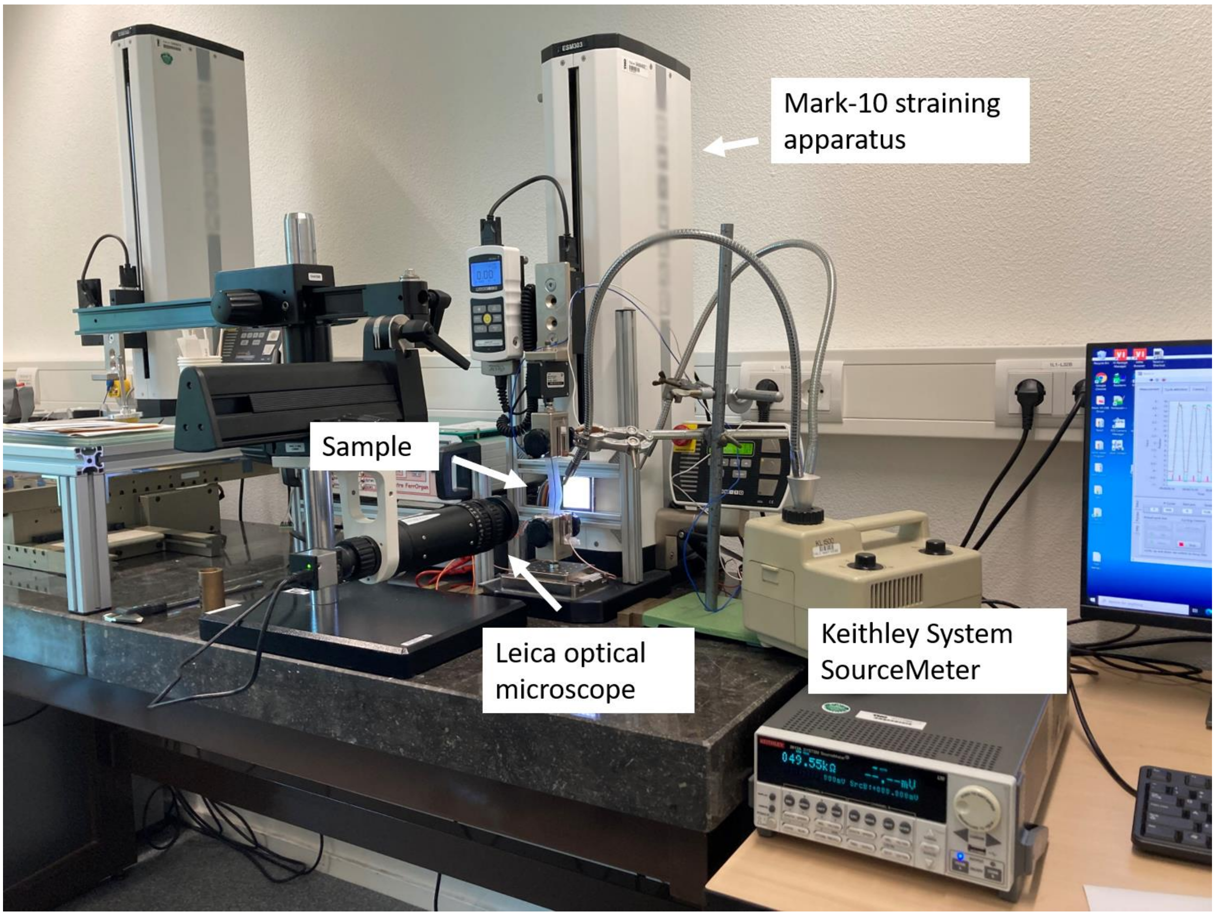

Figure S10. Setup used for electromechanical characterization, including a Mark-10 straining apparatus (Model ESM303), Keithley 2612A System SourceMeter with 4 wires, a Leica Z16 APO microscope and the sample.

9. Strain test with increasing strain amplitude

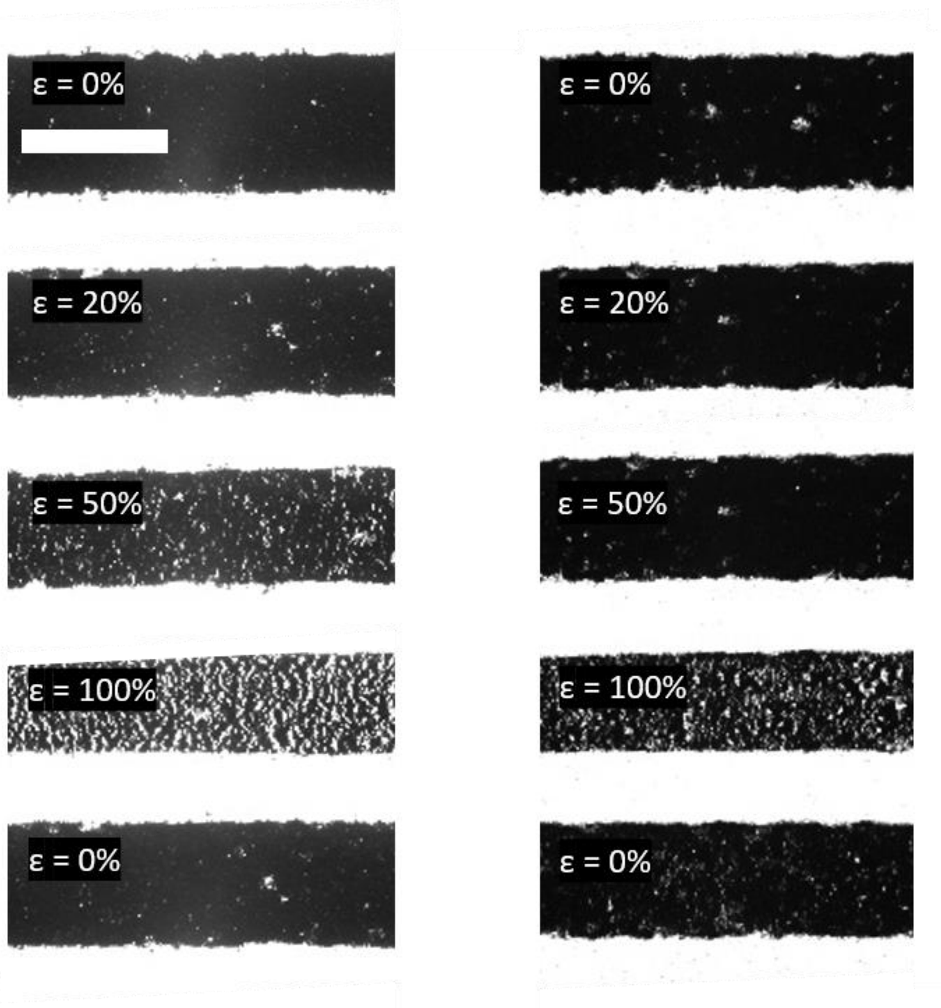

Figure S11. Optical micrographs of printed tracks on EU94 (left) and ST604 (right) before straining (ε = 0%), under uniaxial load (ε = 20, 50, 100%) and after final relaxation. Although the applied strain after final relaxation was 0%, the residual strain was 11.3% for EU94 and 34.0% for ST604.

Table S3. Gauge factors (GF_{ε}) for selected strain levels ε during a strain test with increasing strain amplitude with ε increasing from 2 to 100% and a strain rate of 200 mm min^{-1} (Figure 2).

Sample	GF_{2}	GF_{4}	GF_{10}	GF_{20}	GF_{30}	GF_{40}	GF_{50}	GF_{60}	GF_{70}	GF_{80}	GF_{90}	GF_{100}
EU94	13.1	11.6	12.1	15.1	20.2	28.4	40.7	57.9	82.8	119	171	253
ST604	14.9	13.7	14.3	17.6	23.4	31.9	43.7	61.4	84.7	112	155	234

10. Serpentines vs straight lines: Electromechanical response

Serpentine- or meander-shaped printed tracks are often employed to mitigate the effects of straining on metal-based conductors.[8–14] The high fatigue-resistance and low gauge factors of our straight printed tracks make serpentine-shaped tracks obsolete for the GNP-based conductors studied in the current work. Nonetheless, serpentines could mitigate the rise in resistance of our printed conductors by a factor of almost two as is shown in Figure S12. For technological application, the tracks are sometimes cut before lamination. Cutting results in drastically reduced strain levels, because the cut samples are effectively just being unrolled rather than stretched during the strain test. This does not apply significant strain to the sample. It should be noted that laminated cut samples likely display a behavior intermediate between regular uncut serpentines and the cut versions shown here, tending more towards the unprocessed versions.

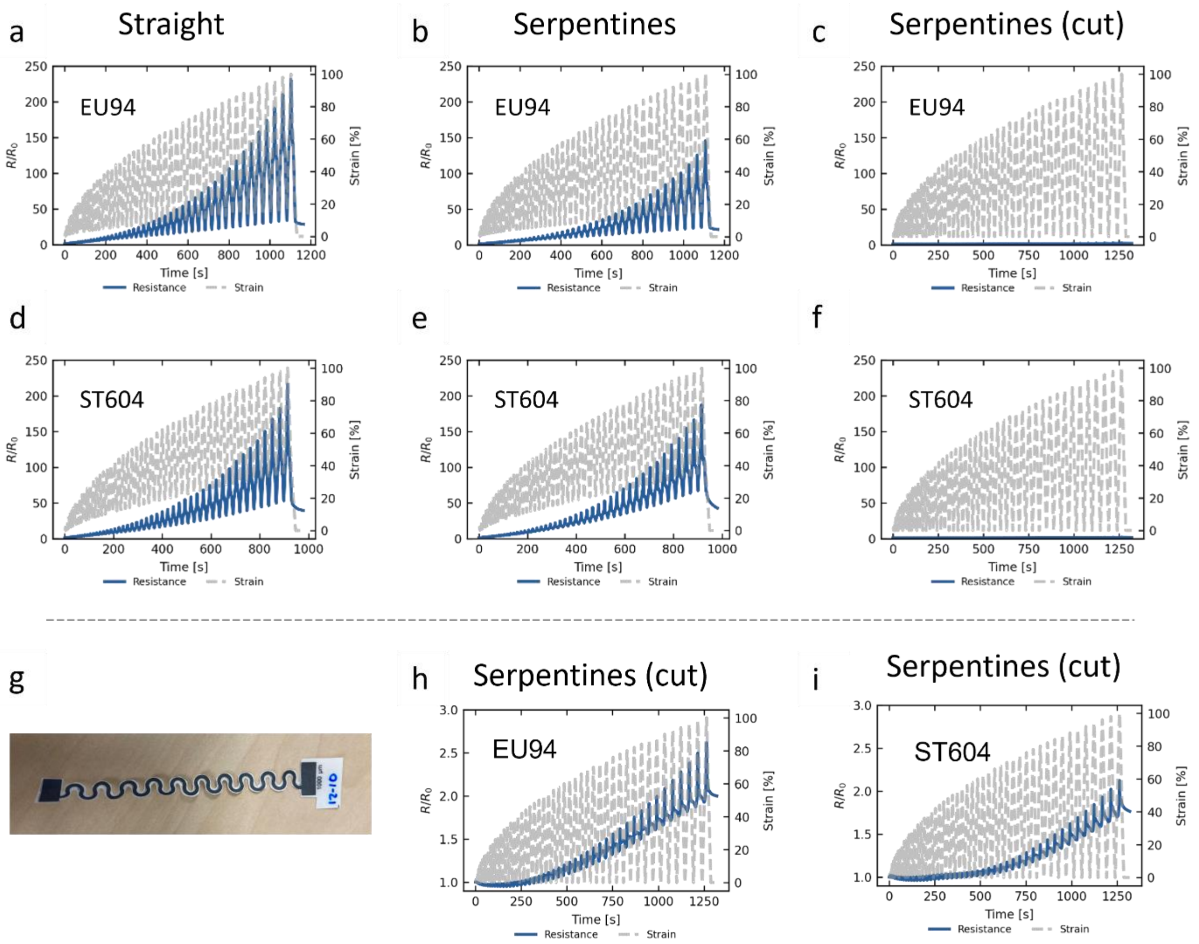

Figure S12. Electromechanical characterization of straight or serpentine-shaped conductors exposed to strains with linearly increasing amplitude from 2-100% in 50 steps with a strain rate of 200 mm min^{-1} on (a-c, h) EU94 or (d-f, i) ST604. The serpentines in panels (c, f, h-i) were cut around the tracks as shown in panel (g).

11. Repetitive strain tests with 20-50% peak strain

11.1 Resistance response

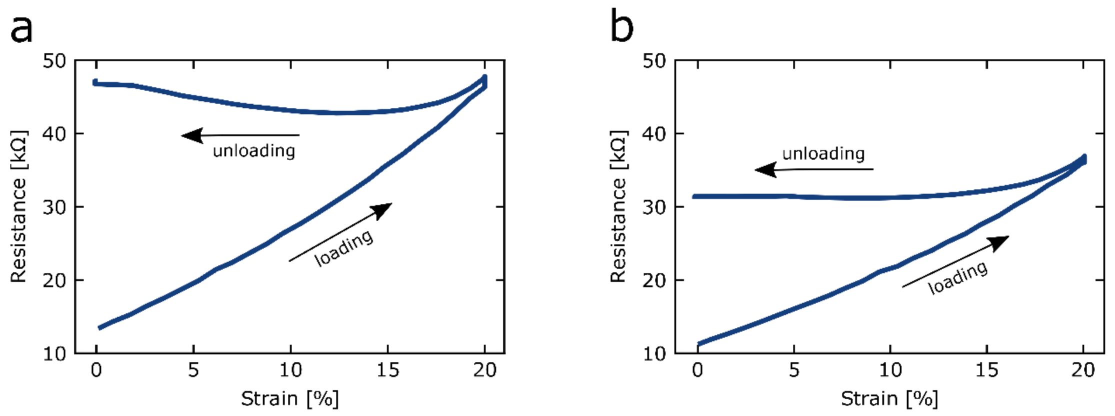

Figure S13. Resistance-strain curves during the first loading and unloading cycle for conductive tracks printed on (a) EU94 and (b) ST604 submitted to a peak strain of 20%

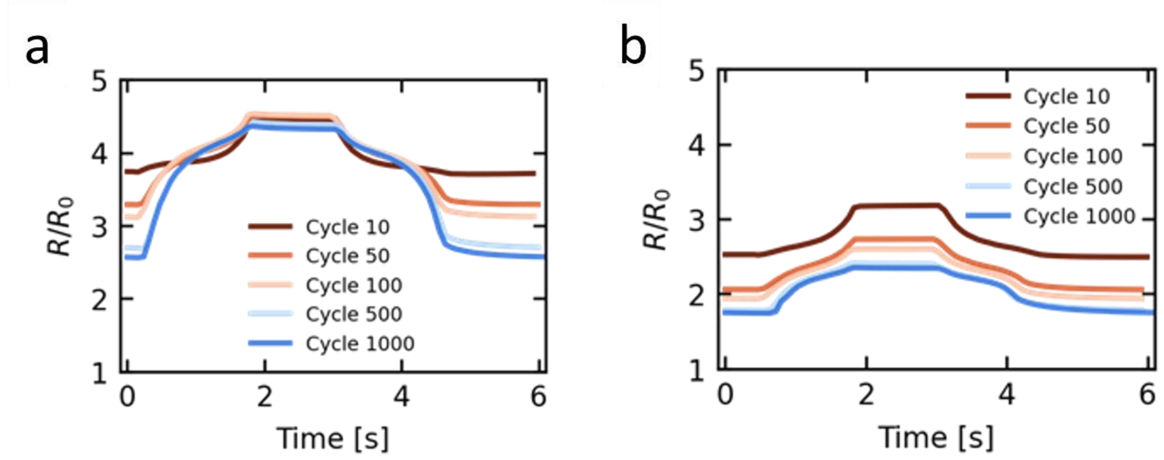

Figure S14. Normalized resistance during selected cycles of cyclic straining at 20% strain of conductors printed on a) EU94 and b) ST604.

11.2 Scanning electron micrographs

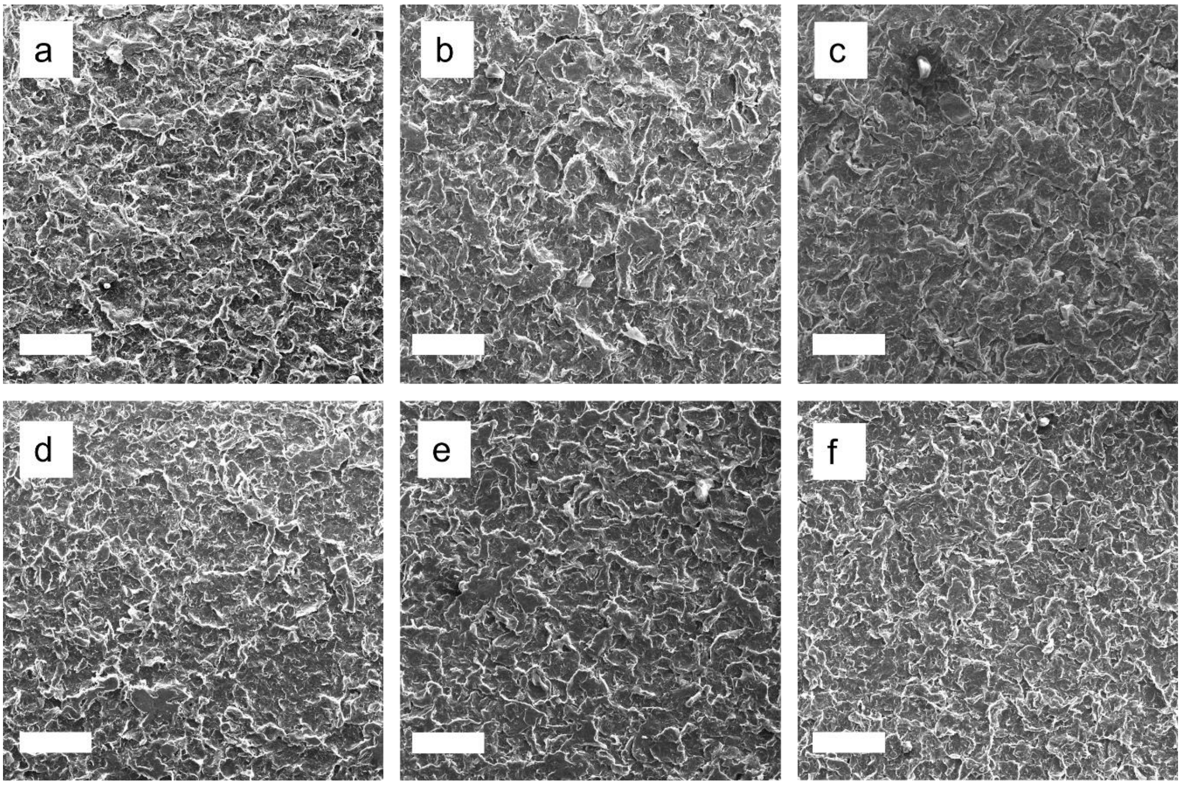

Figure S15. Scanning electron micrographs of conductors printed on (a-c) ST604 and (d-f) EU94 before (a, c) and after (b, e) straining for 1000 cycles of 20% strain with a strain rate of 500 mm/min and (c, f) for 50 cycles with a linear increase in strain amplitude from 2-100% strain and a strain rate of 200 mm/min. These images do not reveal any detectable damage induced by straining. Images were taken at 10 kV with a Phenom ProX SEM equipped with a secondary electron detector (SED). The scale bar represents 200 µm.

11.3 Residual strain of substrates due to rapid cycling

In this study, cyclic straining was performed rapidly (500 mm min^{-1} or 658% min^{-1}) with insufficient time for complete substrate relaxation. Hysteresis and rate dependence of the mechanical behavior are a common phenomenon among thermoplastic polyurethanes.[15] As shown below in Figure S16 and Table S7, this resulted in residual strain (ε_{res}) levels of approximately 3% (EU94) and 8% (ST604) after exposure to cyclic strains of 20%. The substrate hysteresis was observed to increase during cycling, which means that the substrate remains extended. Thanks to this increasing residual strain, the effective strain $\varepsilon_{eff} = 20\% - \varepsilon_{res}$ during cycling reduces over time from 20% in cycle 1 to approximately 17% (EU94) or 12% (ST604) in cycle 1000 (Figure S16c-d). The residual strain was extracted from the dataset for each cycle at the first point in the unloading cycle where the stress was equal to zero. The residual strain remained unaffected by photonic annealing.

This substrate hysteresis may be related to the observed behavior where an increase in residual strain correlates with a decrease in gauge factor (Table 2) and resistance (Figure 3a). In particular for ST604, there appears to be a correlation between the two effects.

Dynamic gauge factor

In the main text, the gauge factor was defined in equation 1:

$$GF_i = \frac{\Delta R_i}{R_0 \epsilon}, \qquad (S1)$$

with $\Delta R_i = R_{max,i} - R_0$, where $R_{max,i}$ represents the resistance at the maximum strain level of loading cycle i, R_0 the initial resistance and ε the maximum tensile strain. For studying the dynamic behavior of the resistance during cycling, the dynamic gauge factor (*DGF*) would be more appropriate.[16] This may be defined as follows:

$$DGF_i = \frac{\Delta R_i}{R_{min,i} \epsilon_{eff,i}}. \qquad (S2)$$

Here, $\Delta R_i = R_{max,i} - R_{min,i}$, with $R_{min,i}$ the resistance at the minimum strain level of loading cycle i, and $\varepsilon_{eff,i} = 20\% - \varepsilon_{res,i}$. The resulting dynamic gauge factors are presented in Table S5.

During cycling, the dynamic gauge factors after the first cycle increase slightly over time but remain very low until they reach a close-to-stable level after cycle 500-800.

As hypothesized previously for stretchable composites of GNPs embedded in a PDMS matrix,[17] and for TPU-GNP composites with very low GNP loading,[18] these low DGFs may be due to rearrangement of GNPs inside the stretchable polyurethane matrix, thereby absorbing the strain while maintaining GNP connectivity. Interestingly, photonic annealing reduces the dynamic gauge factor as it evolves during cycling. We attribute this to a reduction of stretchability of the polyurethane matrix due to heating.

Finally, it should be noted that the dynamic gauge factor is strain rate-dependent. Slower cycling generally reduces the *DGF* and *GF* for TPU-containing conductive composites.[16,18,19] As strain rates in the current study were very high, the *(D)GF*s could likely be reduced by decreasing the cycling rate. Further studies featuring slower strain cycles would be necessary to understand the role of substrate hysteresis and the GNP-TPU network in the evolution of (dynamic) gauge factors and peak resistance during cycling.

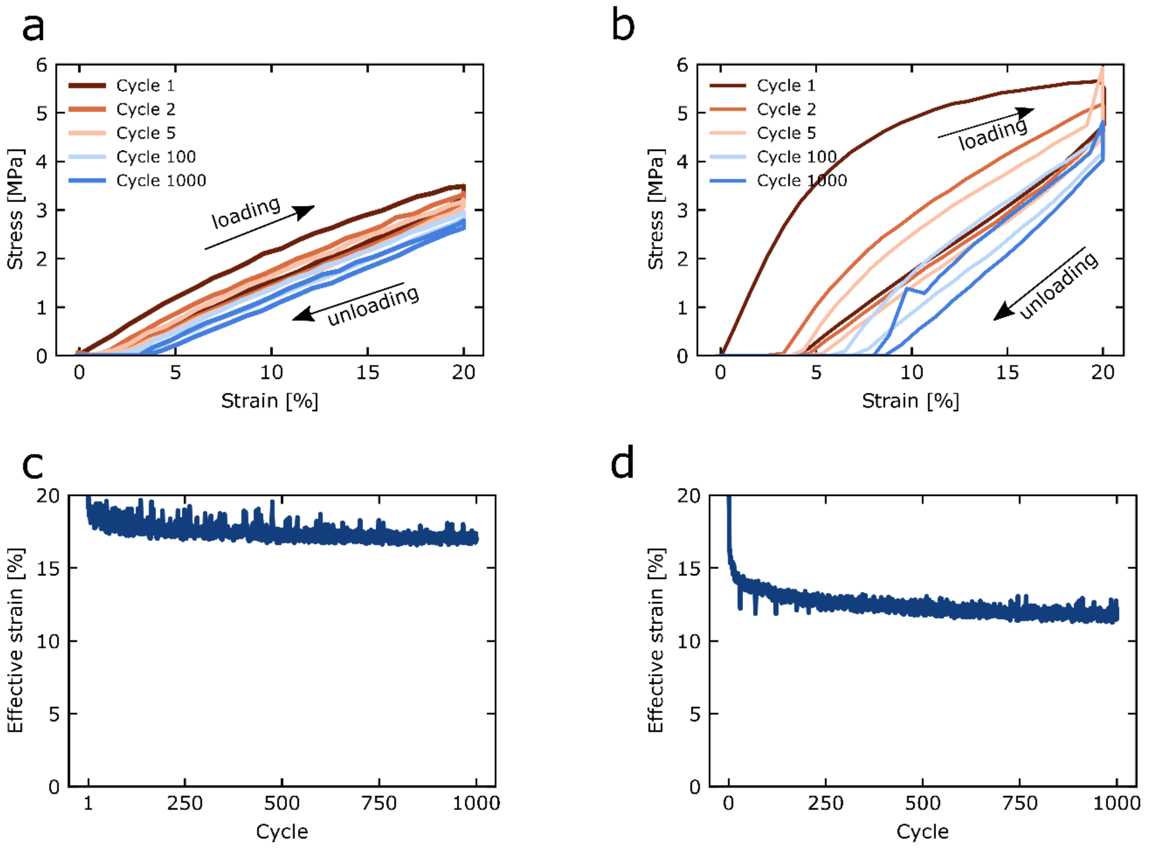

Figure S16. Stress-strain curves during loading and unloading for selected cycles of conductive tracks printed on (a) EU94 and (b) ST604. The intersection with the x-axis indicates the residual strain (ε_{res}) due to substrate hysteresis; c-d) effective strain (ε_{eff} = *20%* - ε_{res}) vs cycle number for conductive tracks on (c) EU94 and (d) ST604.

11.4 Gauge factors

Table S4. Gauge factors (*GF*) during a cyclic strain test with 20% peak strain determined according to equation S1 in cycles *i* = 1, 10, 50, 100, 500, 800 and 1000 for printed conductors on EU94 and ST604 substrates before post-treatment and after photonic annealing with different energy levels indicated by their energy *E* and voltage *V*. Pulse lengths were fixed at 3 ms.

Substrate	*E* [J cm^{-2}]	*i* = 1	*i* = 2	*i* = 10	*i* = 100	*i* = 200	*i* = 500	*i* = 800	*i* = 1000
EU94	Pristine	15.22	18.77	20.70	21.62	21.58	21.09	20.59	20.63
EU94	0.90	11.01	13.46	15.81	17.83	18.73	20.00	20.46	20.28
EU94	1.4	7.36	9.12	12.18	17.23	18.83	20.85	21.82	22.24
EU94	2.3	10.10	12.46	17.56	22.50	23.69	24.99	25.39	25.67
ST604	Pristine	12.29	13.56	12.20	9.06	8.58	8.08	7.96	7.83
ST604	0.62	17.79	21.95	18.90	14.65	13.66	12.30	11.60	11.19
ST604	0.95	9.51	11.46	14.05	16.44	16.83	17.13	17.14	17.13
ST604	1.56	9.58	10.96	13.67	16.81	17.43	18.05	18.20	18.22

Table S5. Dynamic gauge factors (*DGF*) during a cyclic strain test with 20% peak strain determined according to equation S2 in cycles *i* = 1, 10, 50, 100, 500, 800 and 1000 for printed conductors on EU94 and ST604 substrates before post-treatment and after photonic annealing with different energy levels indicated by their energy *E* and voltage *V*. Pulse lengths were fixed at 3 ms.

Substrate	*E* [J cm^{-2}]	*i* = 1	*i* = 2	*i* = 10	*i* = 100	*i* = 200	*i* = 500	*i* = 800	*i* = 1000
EU94	Pristine	12.15	0.81	0.89	2.51	3.04	3.67	3.96	4.08
EU94	0.90	9.63	0.94	0.84	1.81	2.22	2.78	3.11	3.04
EU94	1.4	6.40	1.08	0.52	1.01	1.22	1.52	1.65	1.73
EU94	2.3	9.16	1.15	0.62	0.82	0.94	1.02	1.05	1.06
ST604	Pristine	10.83	1.39	1.61	2.44	2.70	2.77	2.86	2.83
ST604	0.62	15.48	1.15	0.95	1.69	1.80	2.01	2.05	1.97
ST604	0.95	8.47	1.29	0.79	1.44	1.56	1.57	1.66	1.64
ST604	1.56	9.04	0.81	0.51	0.65	0.56	0.74	0.71	0.71

Table S6. (Dynamic) gauge factors ((*D*)*GF*) for selected cycles *i* during a cyclic strain test with 50% peak strain on EU94 (Figure S12)

Sample	*i* = 1	*i* = 2	*i* = 10	*i* = 100	*i* = 200	*i* = 500	*i* = 800	*i* = 1000
GF	23.9	34.7	45.5	51.5	53.0	53.9	54.1	54.3
DGF	23.4	2.21	2.61	5.58	6.31	7.28	7.47	7.62

11.5 Elastic moduli

Table S7. Elastic moduli (E and E_{eff}) and residual strain in cycle 1000 ($\varepsilon_{res,1000}$) for different samples strained for 1000 cycles with 20% strain. Note that two values are missing due to the strain test being aborted during the first cycle due to a software failure. Moduli were extracted from the stress-strain data as explained under Figure S12.

**Substrate**	***E*** **[J/cm²]**	E_{eff} **(unloading) [MPa]**	***E*** **(loading) [MPa]**	$\varepsilon_{res,1000}$ **[%]**
ST604	-	34.2	88.2	7.8
ST604	0.62	33.2	-	8.3
ST604	0.95	34.3	97.7	7.7
ST604	1.56	34.2	-	6.9
EU94	-	15.7	22.6	3.0
EU94	0.90	16.7	25.9	2.5
EU94	1.40	16.7	25.3	2.7
EU94	2.25	16.4	25.0	2.9

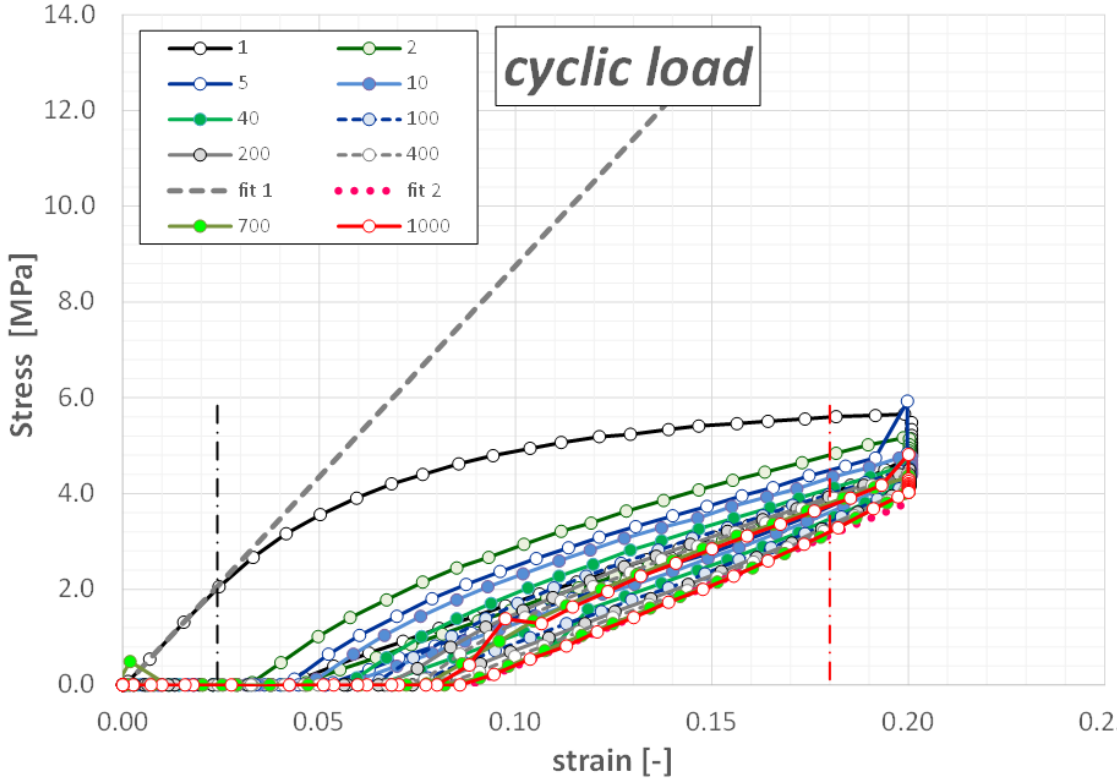

Figure S17. Fit procedure to extract the loading and unloading elastic moduli from the stress-strain curves. The effective Young's moduli are determined from the first loading curve and the last unloading curve based on a least square fit with the solver in Microsoft Excel. It should be noted that the accuracy of the loading modulus is limited due to the limited number of datapoints in the fit procedure.

12 Photonic annealing

Table S8. Sheet resistances after photonic annealing with different energy levels and compression rolling and recovered resistance R_f/R_0 after 300 seconds relaxation after strain cycle 1000 (*N* = 1)

**Substrate**	***E*** **[J cm^{-2}]**	**Compression**	***R$_s$*** **[Ω □$^{-1}$ mil^{-1}]**	***R$_f$/R$_0$***
ST604	-	no	62.9	1.67
ST604	0.62	no	63.1	2.53
ST604	0.62	yes	74.5	1.58
ST604	0.95	no	30.2	3.24
ST604	0.95	yes	45.6	2.43
ST604	1.56	no	13.6	3.98
ST604	1.56	yes	23.5	2.25
EU94	-	no	34.8	2.59
EU94	0.9	no	29.9	3.02
EU94	0.9	yes	29.2	2.67
EU94	1.4	no	9.94	3.77
EU94	1.4	yes	12.2	3.51
EU94	2.25	no	7.01	4.26
EU94	2.25	yes	10.2	3.48

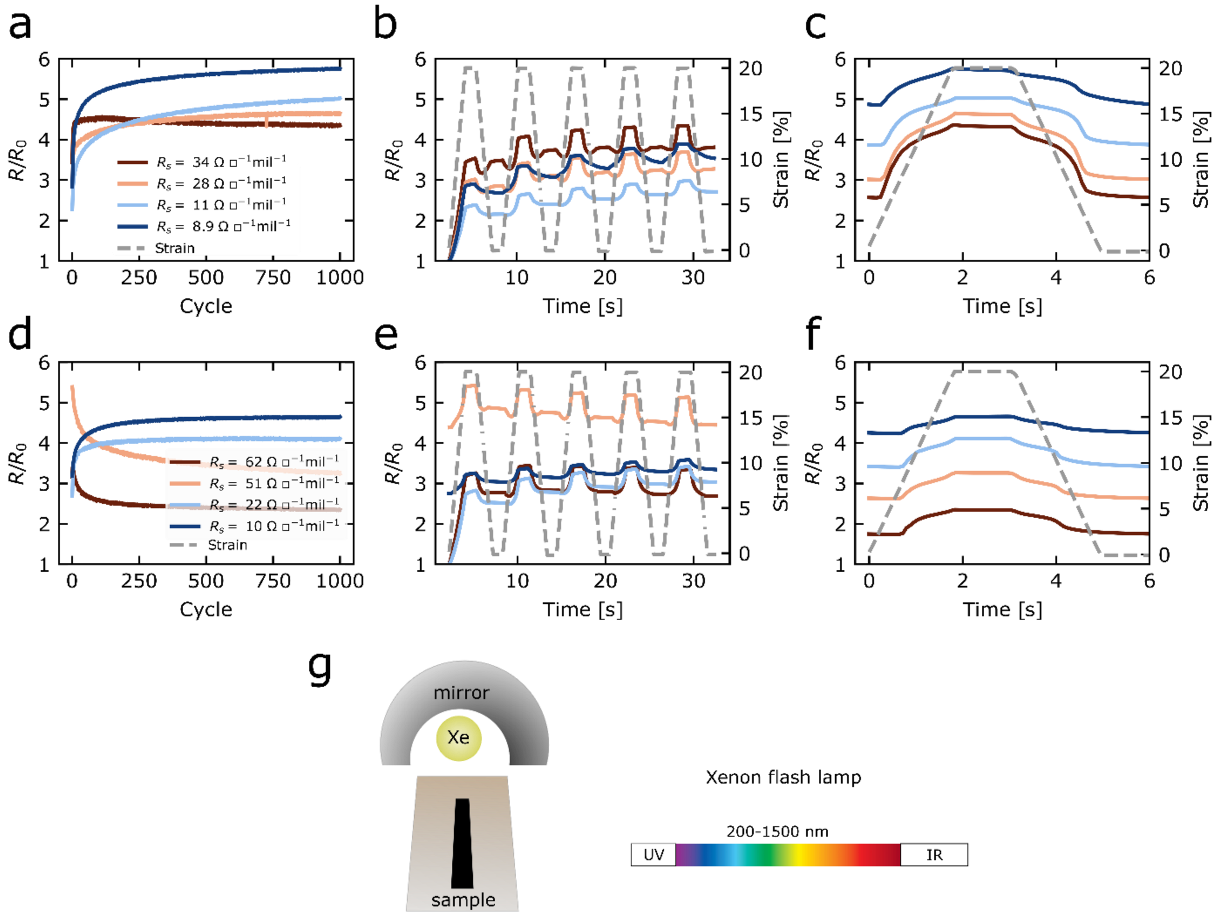

Figure S18. Effect of photonic annealing with increasing energy level on the normalized resistance R/R_0 in response to cyclic straining at 20% peak strain for conductors printed on EU94 (a-c) and ST604 (d-f). a,d) Development of the peak resistance R/R_0 over 1000 cycles; b,c,e,f) R/R_0 and strain during cycle 1-5 (b,e) and cycle 1000 (c,f). R_s values represent the averages derived from Table 1 multiplied by the $R_0/R_{0,p}$ factor in Table 3. For EU94, these resistances were achieved with IPL energies E of 0.90, 1.4 and 2.3 J cm^{-2}, while the applied energies were 0.62, 0.95 and 1.6 J cm^{-2} for ST604. Pristine samples are presented in dark blue. The lines representing E = 0.62 and 1.6 J cm^{-2} in panel (e) do not start at 1, for the strain program got aborted during cycle 1, after which the measurement was restarted effectively in cycle 2; g) schematic representation of the photonic annealing process with a Xenon flash lamp emitting intense bursts of broad-spectrum light with wavelengths between 200 and 1500 nm. A mirror ensures that most light reaches the sample.

13 Wristband

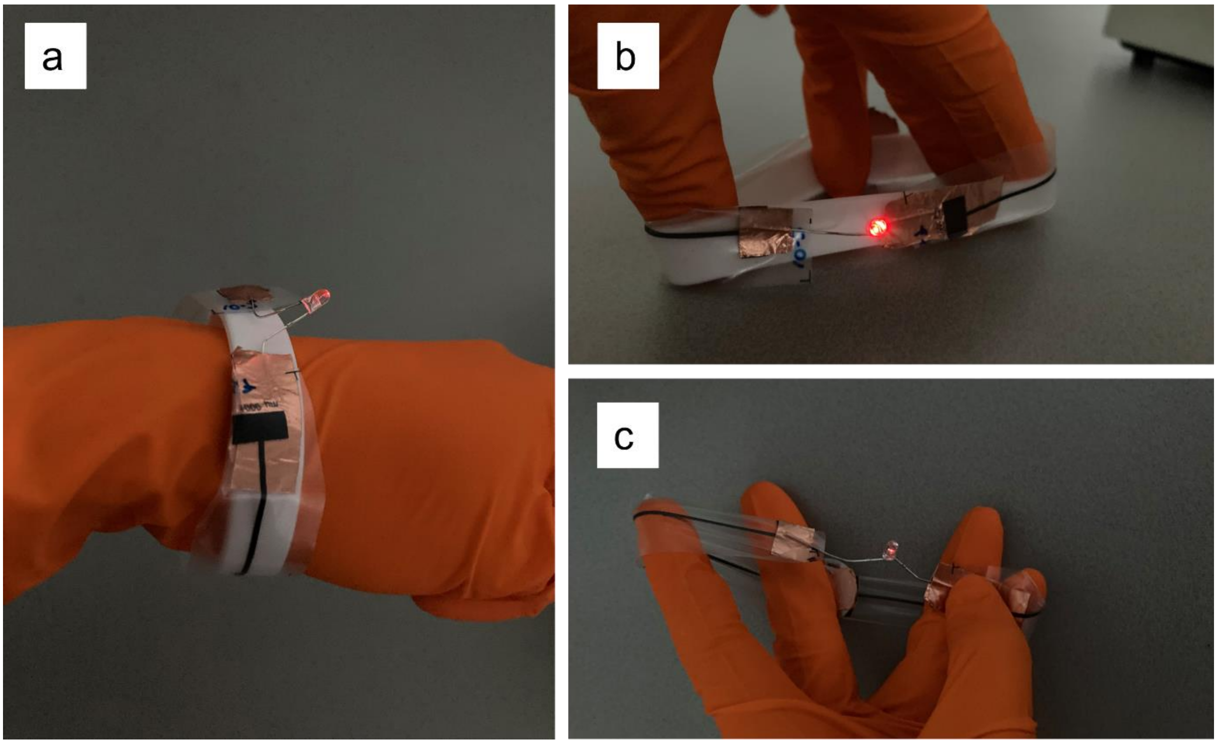

Figure S19. A wearable, stretchable and flexible wristband formed from a circuit composed of two printed and photonically annealed (E = 2.3 J cm^{-2}) GNP tracks on EU94, a red LED (I_F = 20 mA, U_F = 2V) and a 3V lithium Energizer® battery, which were connected with copper tape. Everything was laminated on a silicone bracelet using Neorez U-431 resin; a) wristband worn on the wrist; b) wristband under (limited) strain; c) circuit without silicone bracelet under significant strain.

14 References

(1) Arapov, K.; Rubingh, E.; Abbel, R.; Laven, J.; de With, G.; Friedrich, H. Conductive Screen Printing Inks by Gelation of Graphene Dispersions. *Adv. Funct. Mater.* **2016**, *26* (4), 586–593.

(2) Arapov, K.; Goryachev, A.; de With, G.; Friedrich, H. A Simple and Flexible Route to Large-Area Conductive Transparent Graphene Thin-Films. *Synth. Met.* **2015**, *201*, 67–75.

(3) Ni, Z.; Wang, H.; Kasim, J.; Fan, H.; Yu, T.; Wu, Y. H.; Feng, Y.; Shen, Z. Graphene Thickness Determination Using Reflection and Contrast Spectroscopy. *Nano letters* **2007**, *7* (9), 2758–2763.

(4) Haney, R.; Tran, P.; Trigg, E. B.; Koerner, H.; Dickens, T.; Ramakrishnan, S. Printability and Performance of 3D Conductive Graphite Structures. *Additive Manufacturing* **2021**, *37*, 101618. https://doi.org/10.1016/j.addma.2020.101618.

(5) Claypole, A.; Claypole, J.; Holder, A.; Claypole, T. C.; Kilduff, L. Rheology of High-Aspect-Ratio Nanocarbons Dispersed in a Low-Viscosity Fluid. *J. Coat. Technol. Res.* **2020**, *17* (4), 1003–1012. https://doi.org/10.1007/s11998-020-00319-2.

(6) Ryan, J. A.; Ulrich, J. M. *Quantmod: Quantitative Financial Modelling Framework*; 2020.

(7) Baig, Z.; Mamat, O.; Mustapha, M.; Mumtaz, A.; Munir, K. S.; Sarfraz, M. Investigation of Tip Sonication Effects on Structural Quality of Graphene Nanoplatelets (GNPs) for Superior Solvent Dispersion. *Ultrason. Sonochem.* **2018**, *45*, 133–149. https://doi.org/10.1016/j.ultsonch.2018.03.007.

(8) Bandodkar, A. J.; Nuñez-Flores, R.; Jia, W.; Wang, J. All-Printed Stretchable Electrochemical Devices. *Adv. Mater.* **2015**, *27* (19), 3060–3065. https://doi.org/10.1002/adma.201500768.

(9) Mohammed, A.; Pecht, M. A Stretchable and Screen-Printable Conductive Ink for Stretchable Electronics. *Appl. Phys. Lett.* **2016**, *109* (18), 184101.

(10) Biswas, S.; Reiprich, J.; Pezoldt, J.; Hein, M.; Stauden, T.; Jacobs, H. O. Stress-Adaptive Meander Track for Stretchable Electronics. *Flex. Print. Electron.* **2018**, *3* (3), 032001. https://doi.org/10.1088/2058-8585/aad583.

(11) Yokus, M. A.; Foote, R.; Jur, J. S. Printed Stretchable Interconnects for Smart Garments: Design, Fabrication, and Characterization. *IEEE Sensors J.* **2016**, *16* (22), 7967–7976. https://doi.org/10.1109/JSEN.2016.2605071.

(12) Park, H. J.; Jeong, J.; Son, S. G.; Kim, S. J.; Lee, M.; Kim, H. J.; Jeong, J.; Hwang, S. Y.; Park, J.; Eom, Y.; Choi, B. G. Fluid-Dynamics-Processed Highly Stretchable, Conductive, and Printable Graphene Inks for Real-Time Monitoring Sweat during Stretching Exercise. *Adv. Funct. Mater.* **2021**, *31* (21), 2011059. https://doi.org/10.1002/adfm.202011059.

(13) Jahanshahi, A.; Gonzalez, M.; Brand, J. van den; Bossuyt, F.; Vervust, T.; Verplancke, R.; Vanfleteren, J.; Baets, J. D. Stretchable Circuits with Horseshoe Shaped Conductors Embedded in Elastic Polymers. *Jpn. J. Appl. Phys.* **2013**, *52* (5S1), 05DA18. https://doi.org/10.7567/JJAP.52.05DA18.

(14) Bossuyt, F.; Guenther, J.; Löher, T.; Seckel, M.; Sterken, T.; de Vries, J. Cyclic Endurance Reliability of Stretchable Electronic Substrates. *Microelectron. Reliab.* **2011**, *51* (3), 628–635. https://doi.org/10.1016/j.microrel.2010.09.032.

(15) Qi, H. J.; Boyce, M. C. Stress–Strain Behavior of Thermoplastic Polyurethanes. *Mech. Mater.* **2005**, *37* (8), 817–839. https://doi.org/10.1016/j.mechmat.2004.08.001.

(16) Lynch, P. J.; Ogilvie, S. P.; Large, M. J.; Graf, A. A.; O'Mara, M. A.; Taylor, J.; Salvage, J. P.; Dalton, A. B. Graphene-Based Printable Conductors for Cyclable Strain Sensors on Elastomeric Substrates. *Carbon* **2020**, *169*, 25–31.

(17) Shi, G.; Zhao, Z.; Pai, J.; Lee, I.; Zhang, L.; Stevenson, C.; Ishara, K.; Zhang, R.; Zhu, H.; Ma, J. Highly Sensitive, Wearable, Durable Strain Sensors and Stretchable Conductors Using Graphene/Silicon Rubber Composites. *Adv. Funct. Mater.* **2016**, *26* (42), 7614–7625. https://doi.org/10.1002/adfm.201602619.
(18) Liu, H.; Li, Y.; Dai, K.; Zheng, G.; Liu, C.; Shen, C.; Yan, X.; Guo, J.; Guo, Z. Electrically Conductive Thermoplastic Elastomer Nanocomposites at Ultralow Graphene Loading Levels for Strain Sensor Applications. *J. Mater. Chem. C* **2016**, *4* (1), 157–166.
(19) Wang, Y.; Hao, J.; Huang, Z.; Zheng, G.; Dai, K.; Liu, C.; Shen, C. Flexible Electrically Resistive-Type Strain Sensors Based on Reduced Graphene Oxide-Decorated Electrospun Polymer Fibrous Mats for Human Motion Monitoring. *Carbon* **2018**, *126*, 360–371. https://doi.org/10.1016/j.carbon.2017.10.034.
